# Small or absent Visual Word Form Area is a trait of dyslexia

**DOI:** 10.1101/2025.01.14.632854

**Published:** 2025-01-15

**Authors:** Jamie L. Mitchell, Maya Yablonski, Hannah L. Stone, Mia Fuentes-Jimenez, Megumi E. Takada, Kenny A. Tang, Jasmine E. Tran, Clementine Chou, Jason D. Yeatman

**Affiliations:** 1 Graduate School of Education, Stanford University, Stanford, CA, USA.; 2 Department of Psychology, Stanford University, Stanford, CA, USA.; 3 Division of Developmental-Behavioral Pediatrics, Department of Pediatrics, Stanford University School of Medicine, Stanford,California, USA.; 4 Department of Psychological & Brain Sciences, University of California, Santa Baraba, CA, USA.; 5 Department of Special Education, Peabody College of Education and Human Development, Vanderbilt University, Nashville, TN, USA.; 6 School of Education, University of California, Irvine, CA, USA.

## Abstract

Understanding the balance between plastic and persistent traits in the dyslexic brain is critical for developing effective interventions. This longitudinal intervention study examines the Visual Word Form Area (VWFA) in dyslexic and typical readers, exploring how this key component of the brain’s reading circuitry changes with learning. We found that dyslexic readers show significant differences in VWFA presence, size, and tuning properties compared to typical readers. While reading intervention improved reading skills and increased VWFA size, disparities persisted, suggesting that VWFA abnormalities are an enduring trait of dyslexia. Notably, we found that even with sufficient intervention to close the reading skill gap, dyslexic readers are still expected to have smaller VWFAs. Our results reveal intervention-driven long-term neural and behavioral changes, while also elucidating stable differences in the functional architecture of the dyslexic brain. This provides new insights into the potential and limitations of learning-induced plasticity in the human visual cortex.

## Introduction

The Visual Word Form Area (VWFA) is a region of high-level visual cortex that is tuned to the visual features of written language^1,2^ and is thought to be directly linked to reading ability^3,4^. This text-selective area of cortex is localized to the occipitotemporal sulcus (OTS)^5,6^, in the posterior and lateral portion of left ventral occipitotemporal cortex (VOTC). Because of its close relationship to reading ability, the VWFA is often only detected in literate adults^7^ and children who have already learned how to read^8-10^. Furthermore, VOTC is the most common anatomical region cited in relation to reading disabilities (i.e., dyslexia)^11^. Previous research revealed that people with dyslexia display patterns of underactivation^12-14^ and decreased text-selectivity^9^ in VOTC compared to typical readers. Some suggest that this is due to disruption of typical function in this region for children with dyslexia^15,16^, and that this disruption is due to lack of functional specificity and increased sensitivity to non-text stimuli^17^. However, it remains unclear whether differences in this region are a stable trait of dyslexia or if text-selectivity in VOTC can change as struggling readers improve their reading ability with targeted intervention.

Understanding the dynamic interplay between cortical plasticity and stability is essential for translating research findings into impactful clinical, educational, and policy applications^18^. This understanding is particularly crucial when evaluating the persistence of biological markers even as behavioral improvements are observed. To date, while there has been extensive research on the effects of reading interventions on the brain's reading circuitry^19,20^, gaps remain in understanding which functional properties are malleable and which are stable traits distinguishing dyslexic from typical readers. Recent evidence suggests that effective reading intervention drives structural changes in white matter tracts involved in reading^21,22^. However, findings regarding the intervention-driven changes in the functional properties of VOTC have been mixed. While some studies report that intervention increases VOTC activation, others do not^19^. Further, there are not enough studies that investigate the long term effects of intervention and whether any brain changes are long lasting^23^.

To address these gaps, in the present study we assessed the functional properties of VOTC in a large group of children with dyslexia who participated in a targeted reading intervention. We used dense neuroimaging sampling (up to 5 times over the course of one year) to detail the short- and long-term intervention effects on the functional properties of the VWFA. We use longitudinal modeling to characterize relationships between intervention, VWFA function, and behavioral outcomes of reading ability and ask whether differences in the VWFA are a stable trait of dyslexia or are ameliorated with intervention.

## Results

**Figure 1 ∣:**
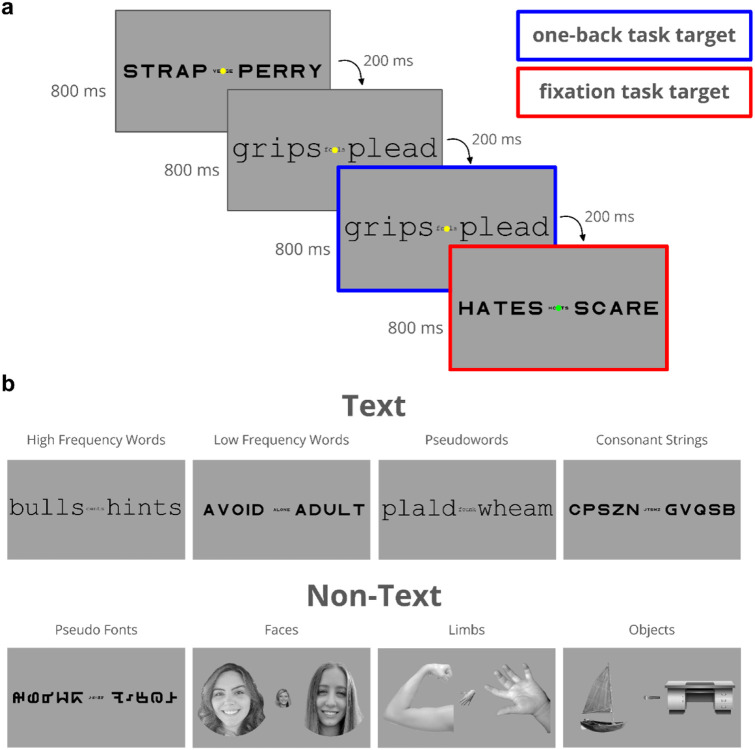
Functional Localizer Experimental Design **a**, Example of a single trial of the experiment. Each frame is presented for 800 ms followed by a blank fixation screen (not shown in figure) for 200 ms. Each trial contains 4 frames of the same stimulus category and lasts for 4 seconds. Targets for the one-back repetition are highlighted in blue and targets for the fixation color change task are highlighted in red (no colors were used in the actual experiment). **b**, Sample stimuli from each sub-category. The text category (top) is comprised of high- and low-frequency words, pseudo words, and consonant strings. The non-text category (bottom) is comprised of pseudo fonts, faces (male and female), limbs (hands, arms, feet, and legs), and objects. Shown in this illustration are faces of co-authors, but the actual experiment presented faces of people unfamiliar to participants.

Forty-four children with dyslexia who participated in a reading intervention, and 46 controls with (*n*=21) and without dyslexia (*n*=25) underwent functional magnetic resonance imaging (fMRI) and behavioral assessments for up to 5 time points over the course of a year. Participants completed an fMRI experiment (Figure 1a) consisting of a child-friendly adaptation of the localizer developed by White et al, ^24^ which examines fMRI responses to high and low frequency words, pseudowords, consonant strings, pseudo fonts, faces, limbs and objects under different task demands (see Stone et al.^25^ for more details on the experimental design).

**Fig. 2 ∣:**
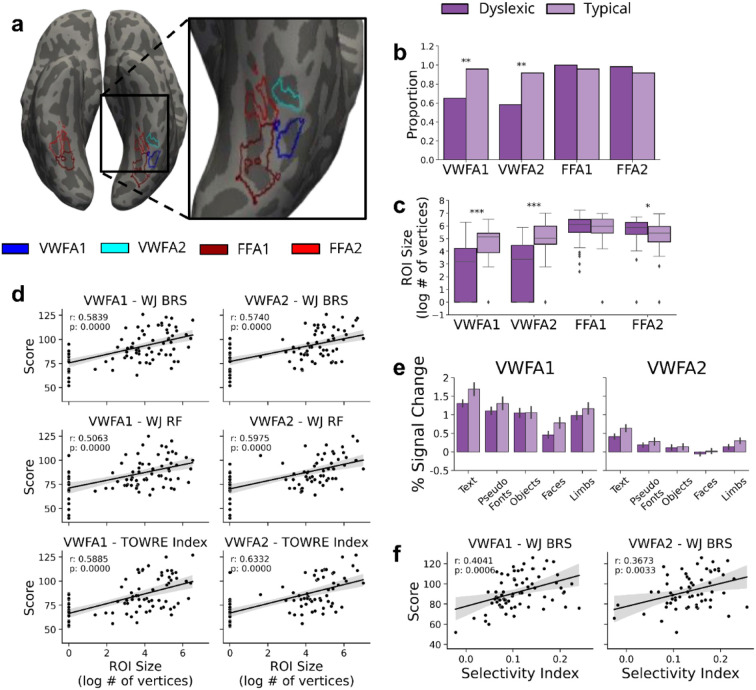
Visual Word Form Area Size and Selectivity Is Related to Reading Ability at Baseline **a**, Example regions of interest (ROIs) on the inflated surface of one example subject. Visual Word Form Area 1 (VWFA-1; blue) & VWFA-2 (cyan) fall on the left occipitotemporal sulcus (OTS) and Fusiform Face Area 1 (FFA-1; dark red) & FFA-2 (red) fall on the fusiform gyrus. **b**, Proportion of participants with usable data who had a VWFA or FFA of any size present at baseline in typical (light purple) and dyslexic (purple) readers for each ROI. **c**, Log transformed size (in number of vertices) at baseline in typical and dyslexic readers for each ROI. **d**, VWFA size is positively correlated with reading tests: Woodcock-Johnson Basic Reading Skill score (WJ BRS; top row), Reading Fluency score (WJ RF; middle row), and Test of Word Reading Efficiency Index (TOWRE index; bottom row). **e**, Percent signal change for each category of visual stimuli (text, pseudo fonts, objects, face, & limbs), within VWFA-1 (left) and VWFA-2 (right). Error bars represent the standard error. **f**, Correlation between selectivity index in VWFA-1 (left) and VWFA-2 (right), and BRS scores across all participants.

Left and right Visual Word Form Area (VWFA) 1 and 2 (posterior to anterior) and Fusiform Face Area (FFA) 1 and 2 (posterior to anterior) were defined on the native cortical surface (see Figure 2a) of each participant at every time point based on the text>non-text and faces>other contrasts (threshold of t>3 within the appropriate anatomical boundaries (see Methods for exclusion criteria). For longitudinal analyses of functional selectivity, additional ROIs were defined by combining all timepoints available for each subject, to increase SNR as well as ensure a consistent ROI across timepoints. To determine any differences in these functionally-defined ROIs, we compared the presence, size, and functional selectivity of each ROI both at the baseline across participants with different levels of reading ability, and longitudinally as reading skills improved.

### VWFA Presence, Size, and Selectivity is related to Reading Ability

We first tested the hypothesis that the VWFA is more likely to be absent (or harder to detect) in children with dyslexia. To this end, we first focused on the baseline (pre-intervention) time point, and compared all participants with dyslexia (*n*=59) to all typical readers (*n*=24). A χ2 test of independence revealed that, compared to typical readers, a smaller proportion of dyslexic participants had a detectable VWFA-1 (dys=65%, typ=95.83%; χ2 = 6.911, p = 0.0086) and VWFA-2 (dys=58.33%, typ=91.66%; χ2 = 7.2715, p = 0.0070) at baseline (Figure 2b). There was no difference in the presence of FFAs between dyslexic and typical readers at baseline (p > 0.20) confirming the specific role of the VWFA as opposed to a general difference extending across VOTC regions.

We next tested the hypothesis that the VWFA is smaller in participants with dyslexia compared to typical controls. A t-test revealed that participants with dyslexia had significantly smaller ROIs than typical readers for both VWFA-1 (mean dys= 59.45 vertices, mean typ = 189.54 vertices; *t* = −4.5125, p = 0.000) and VWFA-2 (mean dys= 63.27, mean typ = 285.04; *t* = −4.5060, p = 0.000) but not for either FFA-1 (mean dys= 471.60, mean typ = 471.42; *t* = 0.378, p = 0.706) or FFA-2 (mean dys= 362.33, mean typ = 275.88; *t* = 2.262, p = 0.026, see Figure 2c). To better understand the relationship between VWFA size and reading ability, we next calculated the correlation between ROI size and reading ability as a continuous measure. We used three different reading assessments to capture different aspects of reading: Woodcock-Johnson Basic Reading Skills score (WJ BRS), Woodcock-Johnson Reading Fluency score (WJ RF), and Test of Word Reading Efficiency index (TOWRE Index). We found that the size of both VWFA-1 and VWFA-2 was positively correlated with reading ability for each of the three reading measures (Figure 2d). In contrast, there was no significant correlation between reading ability and FFA size (all p > 0.1). To ensure that this relationship was not driven solely by the individuals who were missing a VWFA (i.e., participants with ROI size of 0), we re-ran the analysis excluding these individuals and found that the significant relationship persisted even within the smaller sample (Supplementary Figure S1).

We then tested the hypothesis that neural tuning properties of the VWFA differ in participants with dyslexia compared to typical controls. Specifically, we calculated response amplitudes to each stimulus condition within each participant’s individually-defined, VWFA ROIs. To determine any difference in activation we ran a linear mixed effects model (LME) looking at the interaction between group and stimulus category, (Supplementary Table S1). We found a group effect (dyslexic < typical) on the mean percent signal change to text in VWFA-1 (β = 0.3687, p = 0.0401), and to a lesser extent in VWFA-2 (β = 0.2060, p = 0.0553; Figure 2e), suggesting that dyslexic participants have weaker text-evoked activation in these regions compared to typical peers. Interestingly, there was a significant interaction showing that the difference between text activation and object activation was larger in the typical reader group compared with the dyslexic group (VWFA-1: β = −0.3750, p = 0.0004; VWFA-2: β = −0.1993, p = 0.0014). This suggests that children with dyslexia have relatively stronger responses to objects, in line with Kubota et al.^9^, in addition to weaker responses to text.

We then calculated a selectivity index for each participant in the sample (Supplementary Table S2), defined as the difference between activation to text versus non text, divided by the sum of activation to all stimuli^9^. We found a significant positive relationship between all the reading scores and selectivity in VWFA-1 (WJ BRS: *r* = 0.404, p = 0.001; WJ RF: *r* = 0.375, p = 0.002; TOWRE: *r* = 0.345, p = 0.005) and with selectivity in VWFA-2 (WJ BRS: *r* = 0.3673, p = 0.0033; WJ RF: *r* = 0.038, p = 0.003; TOWRE: *r* = 0.384, p = 0.003; Figure 2f). This suggests that better reading is associated with greater selectivity for text in the VWFA.

### Reading Intervention Drives Changes in VWFA

**Figure 3 ∣:**
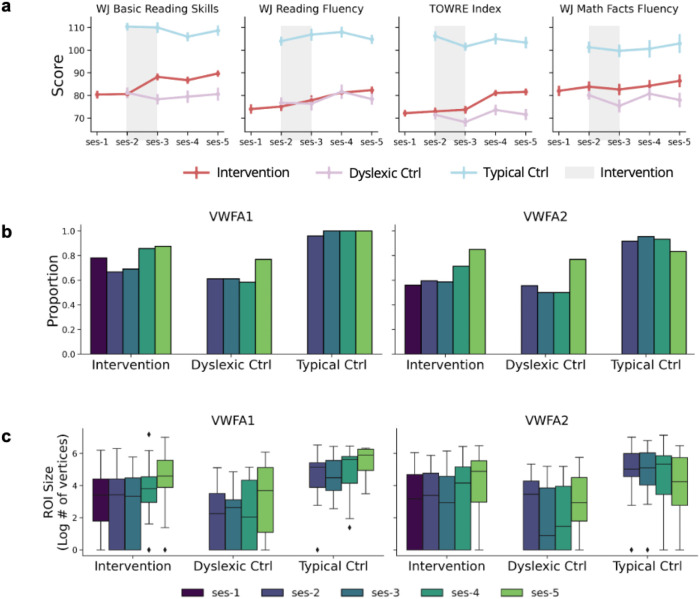
Change in Reading and VWFA **a**, Change in standard score for various reading assessments (Woodcock-Johnson Basic Reading Skills - WJ BRS; Woodcock-Johnson Reading Fluency - WJ RF; and Test of Word Reading Efficiency - TOWRE) and a control math assessment in all three groups of participants. **b**, Proportion of participants with usable data who had an ROI of any size present at every time point in each group for VWFA-1 (left) and VWFA-2 (right). **c**, Size (log transformed number of vertices) at every time point in each group for VWFA-1 (left) and VWFA-2 (right).

To first determine if reading ability improved as a result of the intervention, we fit a LME looking at assessment score as a function of time (in days from baseline) with a random intercept by participant for each study group (Supplementary Table S3). We first confirmed that the reading intervention successfully improved reading ability in the intervention group (WJ BRS: β = 0.021, p = 0.000; WJ RF: *β* = 0.019, p = 0.000; TOWRE: *β* = 0.025, p = 0.000 - Figure 3a). As expected, the dyslexic control group showed no changes in any scores (WJ BRS: β = −0.004, p = 0.423; WJ RF: β = 0.009, p = 0.092; TOWRE: β = 0.004, p = 0.508). Interestingly, the typical control group did show an increase in WJ RF (β = 0.021, p = 0.000) while displaying no significant gains in any other reading assessment (p > 0.2). These results were further supported by a second model fit with the same parameters along with a group interaction. This interaction model confirmed large increases in reading score across all measures for the intervention group (p<0.0001) and significantly smaller rates of change in score for the control groups for WJ BRS (Dys Ctrl: *β* = −0.0248, p = 0.0002; Typ Ctrl: *β* = −0.0263, p = 0.0001) and TOWRE (Dys Ctrl: *β* = −0.0218, p = 0.0002; Typ Ctrl: *β* = −0.0290, p = 0.0000). This significant interaction suggests that the reading improvement was specifically driven by the intervention itself. In contrast, reading fluency and math (WJ Math Facts Fluency - WJ MFF) improved over time, but this effect was not specific to the intervention group (WJ RF: p > 0.0500; WJ MFF: p > 0.1000). After establishing that the intervention was effective in improving reading skills in the intervention group alone, we next tested the hypothesis that this reading improvement drives plasticity in VWFA.

We first determined if the proportion of VWFAs increased after the intervention in children with dyslexia. To do so, we fit generalized linear mixed effects (GLME) models (binomial family for a dichotomous outcome measure) within each group (intervention, dyslexic control, and typical control), predicting the presence of a VWFA ROI as a function of time (in days from baseline). We also included regressors of age (at baseline visit), movement (mean framewise displacement in each session), and number of runs per session (after exclusion for data quality, see Methods) to control for effects of data quality or developmental factors (unless otherwise specified all models include these covariates). Using this approach, we found that the intervention significantly increased the probability of detecting VWFA-1 (β = 0.0044, p = 0.0295) and VWFA-2 (β = 0.0089, p = 0.0011). Since nearly every participant in the typical control group had a well-defined VWFA from the beginning of the study, we could not fit longitudinal GLMER models to the control group (the same is true for the FFA which was present at nearly all time points in all subjects).

To examine changes in VWFA size, we ran a LME where we estimated the size of each ROI (in log transformed number of vertices) as a function of the interaction between group and time with a random intercept for participant, treating the intervention group as the reference (Figure 3C; Supplementary Table S4). We found that the ROI size increased with time in the intervention group for both VWFA-1 (β = 0.0032, p = 3.87e^−7^; ~0.321 vertices per day) and VWFA-2 (β = 0.0032, p = 3.73e^−7^; ~0.245 vertices) while there was a negative interaction effect with time for the typical control group in VWFA-2 (β = −0.0049, p = 0.0003; ), indicating that the typical control group did not show growth in VWFA-2. Furthermore, a main effect of group revealed that the dyslexic controls had a smaller VWFA-1 (β = −0.9232, p = 0.0340) while the typical controls had a larger VWFA-1 (β = 1.6957, p = 3.97e^−5^) and VWFA-2 (β = 2.3656, p = 5.78e^−6^) over time compared to the intervention group. This suggests that the dyslexic control group has a marginally smaller starting VWFA-1 size compared to the intervention group while the typical control group has significantly larger starting VWFAs than the intervention group, once again confirming that dyslexic readers have smaller VWFAs than typical readers. Interestingly, typical controls showed a negative interaction with time in size of VWFA-2 (β = −0.0049, p=0.0003), indicating that the rate of growth is significantly lower than the intervention group. Finally, no size changes over time were observed in control FFA regions (see Supplementary Table S4).

**Figure 4 ∣:**
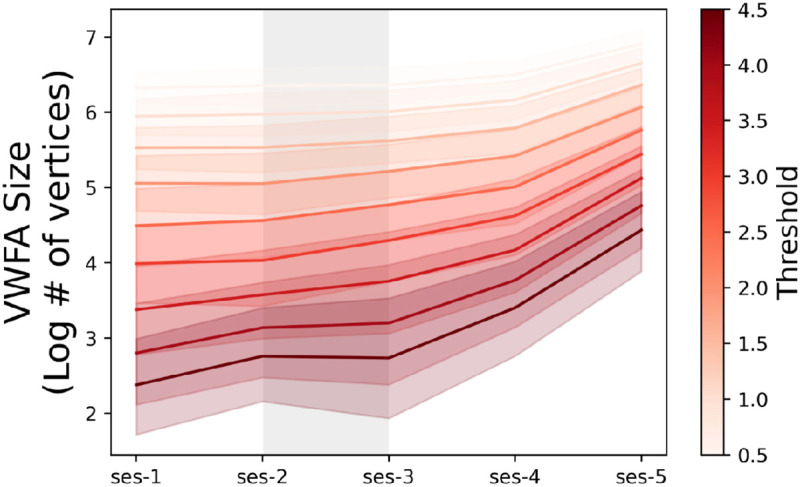
VWFA Shows Growth at Several Thresholds Change in size of VWFA in the intervention participants (*n*=44). For the purposes of this analysis, VWFA was defined as any vertex in the VOTC that had a *t* value >= the threshold value in a contrast map comparing activation to text > activation to all other categories. Thresholds were tested in increments of 0.5 ranging from 0.5 to 4.5.

To ensure that changes in VWFA size were truly the result of a change in tuning properties of the VOTC and not simply the effect of methodological decisions such as the threshold used to define the ROIs, we ran additional analyses on VWFA size at various thresholds. To accomplish this, we drew a large anatomical VOTC ROI in fsaverage template space, projected this label onto the native cortical surface for each intervention participant, and used this label to constrain text-selective contrast maps. We then selected all vertices within these VOTC-masked contrast maps that had a *t* value greater than or equal to different threshold values, in increments of 0.5 ranging from 0.5 to 4.5. Figure 4 shows that regardless of the chosen threshold, intervention participants did in fact show an increase in the number of text-selective vertices in VOTC over time. Furthermore, to ensure that the relationship between reading ability and VWFA size was also maintained across thresholds, we repeated the analysis visualized in Figure 2d and computed correlations between pre-intervention VWFA size and assessment score for each threshold (Supplementary Figure S2). We found that the relationship between reading assessment score and VWFA was still highly correlated at every threshold.

### VWFA Differences Persist After Intervention

We sought to determine if the differences that existed between typical and dyslexic readers at baseline persisted after the conclusion of the intervention, despite confirmed increases in the presence and size of VWFA in the intervention group. We fit a linear mixed effects model looking at percent signal change as a function of the interaction between group, time, and visual category, with a random intercept of participant (Supplementary Table S5). This model revealed that in VWFA-1, typical readers had a greater magnitude of percent signal change to text than intervention participants (*β = 0.3809,* p = 0.0192), and that the difference between activation to text and non-text stimuli was greater for typical readers than intervention participants (*β = −0.1830,* p = 0.0243). There were no changes in activations as a result of the intervention in VWFA-1 (*β = 0.0002,* p = 0.1731), suggesting that the group effect observed at baseline persists despite intervention in VWFA-1. In VWFA-2, the intervention group showed increased signal to text over time (*β* = 0.0003, p = 0.0086), mirroring findings from previous work^26^. To determine if the elevated object sensitivity in VWFA-2 for dyslexic readers seen at baseline persisted after intervention, we ran a post-hoc analysis comparing mean percent signal change from baseline (time point 2) to the final time point (time point 5). A paired samples t-test revealed that while response to text increased significantly in the intervention group (t = 3.137, p = 0.004, df=29), responses to objects only marginally increased (t = 2.137, p = 0.0417).

To further investigate any longitudinal change in tuning properties of the VWFA, we ran another LME looking at text selectivity index as a function of the interaction between time and group (Supplementary Table S6). We found that text selectivity increased in the intervention group in VWFA-1 (*β* = 0.0001, p = 0.0202) but not in VWFA-2 (*β* = 0.0001, p = 0.0717). We also found no effect of group nor an interaction effect of group and time indicating that the trajectory of selectivity index change over time was not significantly different in the intervention group in comparison to the control groups.

### Visual Word Form Area size tracks growth in reading ability

After determining that VWFA size changes with time, we next sought to investigate whether this growth is coupled with reading improvement. To this end, we calculated a reading “trait” score for each assessment for every participant by computing an average score across all available time points. We next calculated a reading “state” score by subtracting each participant’s trait score from their score in each time point. We use trait and state scores to tease apart the effects of within participant change in score from the between-participant individual differences (captured by the “trait” scores)^27^. We then ran a linear mixed effects model looking at ROI size as a function of reading trait and the interaction between reading state and study group, with a random intercept of participant (Table 1). This analysis revealed a main effect of reading trait across all reading assessments in both VWFA-1 (WJ BRS: *β* = 0.0482, p = 0.0049; WJ RF: *β* = 0.0287, p = 0.0414; TOWRE: *β* = 0.0562, p = 0.0020) and VWFA-2 (WJ BRS: *β* = 0.0828, p = 0.0001; WJ RF: *β* = 0.0663, p = 0.0001; TOWRE: *β* = 0.1165, p = 7.82e^−8^). This was not the case for the control math assessment (VWFA-1: *β* = −0.0020, p = 0.8585; VWFA-2: *β* = 0.0179, p = 0.2225). This corroborates our findings regarding the pre-intervention time point and further supports our hypothesis that a small VWFA is related to weaker reading ability. The model also revealed a main effect of reading state across all reading assessments on both VWFA-1 size (WJ BRS: *β* = 0.0530, p = 0.0007; WJ RF: *β* = 0.0854, p = 1.98e^−6^; TOWRE: *β* = 0.0389, p = 0.0213) and VWFA-2 size (WJ BRS: *β* =0.0696, p = 5.18e^−6^; WJ RF: *β* = 0.1106, p = 2.24e^−10^; TOWRE: *β* = 0.0575, p = 0.0003). This suggests that a greater increase in reading ability is related to a greater increase in VWFA size. Surprisingly, for WJ RF, dyslexic controls displayed a weaker relationship between reading trait and VWFA-1 size (*β* = −0.9046, p = 0.0223). Additionally, for WJ RF, there was a weaker relationship between change in reading state and change in ROI size for VWFA-2 (*β* = −0.0937, p = 0.0161) for dyslexic controls and in both VWFA-1 (*β* = −0.0865, p = 0.0189) and VWFA-2 (*β* = −0.1381, p = 0.0001) for typical controls. This suggests that change in reading fluency may have a greater relationship to change in VWFA size than our other reading measures. Once again, no relationship was observed between VWFA size state and our control math assessment (VWFA-1: *β* = 0.0165, p = 0.4070; VWFA-2: *β* = 0.0098, p = 0.6176). Furthermore, there were no significant relationships between any reading assessment state or trait on the size of either FFA, confirming that reading ability is specifically associated with VWFA size, rather than driving a general change in other high level visually selective regions. These findings were maintained when re-running models on individual assessment raw scores as opposed to composite standard scores (Supplementary Table S7).

**Figure 6 ∣:**
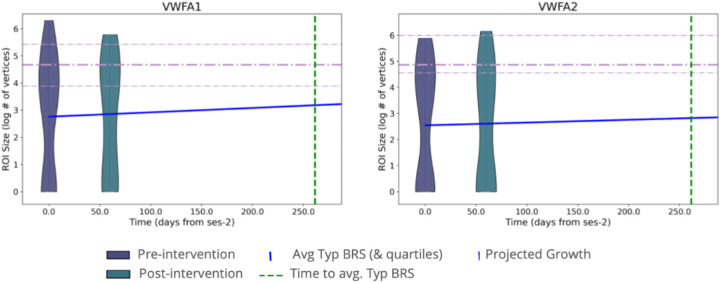
Predicted VWFA size after sufficient intervention Predicted growth of VWFA-1 (left) and VWFA-2 (right) over time with sufficient time of intervention (255 days; dashed green line) to increase BRS scores to the average typical control BRS score assuming linear growth. Violins show the distribution of sizes of VWFAs in the intervention group, in the pre- and post-intervention time points. Dashed purple lines represent the average size (and 25th and 75th percentiles) of a VWFA in the typical control group.

Finally, to extend our investigation of the relationship between VWFA size and dyslexia, we ran an additional analysis to determine what would be the expected VWFA size given an intervention dosage theoretically sufficient to bring a child’s scores to typical reading levels. This analysis included two steps: we first calculated what is the predicted amount of intervention needed to drive an individual child with dyslexia to achieve reading ability comparable to typical readers in our sample. Then, we examined what the projected VWFA size would be given this duration of intervention. For this analysis we assume a linear effect of intervention duration on reading improvement, based on previous findings ^28^. To do this, we first calculate the average WJ BRS score and the average VWFA size for the typical control group at the pre-intervention time point. We then fit a LME to all intervention participants looking at WJ BRS score as a function of time with a random intercept of participant, using data from the pre- and post-intervention time points. Using the result coefficient for time (*β* = 0.1031, p = 6.39e^−05^) and the average typical and average dyslexic BRS scores, we calculated the predicted time (in number of days of intervention) needed for a dyslexic participant to increase their scores to match the average typical reader’s BRS score. In doing so, we found that dyslexic participants would need 255 days of intervention to reach the average score of typical readers - much longer than the 40 days offered in this study. Finally, we fit a second LME to the pre- and post-intervention time points for the dyslexic readers looking at VWFA size as a function of days of intervention. Using these results, we can see that, even with the appropriate amount of intervention to close the gap between dyslexic and typical reader assessment scores, dyslexic readers are still expected to have a smaller VWFA (VWFA-1: 3.1701 log # of vertices, VWFA-2: 2.8113) compared with typical readers (VWFA-1: 4.6344, VWFA-2: 4.8556; Figure 6).

## Discussion

Dyslexia research has long debated which components of the brain’s reading circuitry are plastic and dynamically change with improvements in reading skills, and which (if any) are stable traits that differentiate individuals with dyslexia even after successful intervention. Here, we found both plasticity and stability in the Visual Word Form Area (VWFA). On the one hand, the size and neural tuning properties of the VWFA did change with learning. On the other hand, dramatic differences remained between individuals with dyslexia and typical readers after the intervention, and are projected to remain even if the intervention were extended in length. Based on these findings, we conclude that differences in the VWFA are likely a persistent trait that underlies the continual challenges in reading many children with dyslexia face.

In this study, we combined several techniques to measure the nature of visual word form-evoked responses in left ventral occipitotemporal cortex (VOTC) in a longitudinal sample of control and intervention participants. The current findings highlight significant differences in the existence, size, and functional tuning properties of the VWFA between dyslexic and typical readers. Consistent with previous cross-sectional research, our results confirm that dyslexic readers are less likely to have a detectable VWFA and that this region is smaller compared to their typical peers^7-9^. The reduced detection rates suggest that this region is less specialized for text processing in children with reading difficulties. The significant differences in VWFA size between dyslexic and typical readers further reinforce this notion, as smaller VWFA regions in dyslexic readers may reflect less specialization and efficiency in processing visual word forms. Together, this aligns with the hypothesis that dyslexia is associated with underdeveloped neural substrates critical for fluent reading. Lastly, the positive correlation between VWFA size and reading ability, across all three reading assessments used here, underscores the functional importance of these regions in supporting literacy. This relationship suggests that a more developed VWFA, as reflected by larger ROI sizes, is associated with better reading performance, potentially due to greater neural resources being allocated to process written language. Importantly, we found that these differences persisted even after intervention, indicating that VWFA size and tuning are not merely reflections of reading experience per se, but are also underlying traits of dyslexia. This novel finding suggests that abnormalities in VWFA are enduring characteristics, which likely contribute to the persistent challenges with automating word recognition observed in dyslexic individuals despite improvements in behavioral measures.

Tuning properties of the VWFA also appear to be related to changes in reading ability. As demonstrated in previous studies^9^, text selectivity, or the degree to which response to text is higher than responses to other visual categories, is highly correlated with reading ability. However, previous research has had mixed findings on whether this relationship is due to general hypoactivation of the VWFA^11,12^ as opposed to elevated responses to other categories for dyslexic readers relative to typical readers^9,29^. Our results suggest that the differences in tuning properties between typical and dyslexic readers may be due to a combination of both aspects. Specifically, our data revealed weaker text-evoked activation in VWFA for dyslexic participants compared to their typical peers, in parallel with a stronger response to visual objects. Crucially, these functional differences were not widespread throughout the entire VOTC as previous research suggested^12^. Rather, this response was specific to the VWFA and was not observed in the adjacent Fusiform Face Area (FFA). This further emphasizes the specialized role of the VWFA in reading.

Our longitudinal analyses shed further light on the nature of change in VWFA driven by improved reading ability. As expected, the intervention successfully improved reading ability across several assessments, consistent with previous studies^28^. Several longitudinal studies have shown that VWFA emerges and develops as children learn to read through the school years^30-32^. Here, we found that this process can be accelerated by an intensive intervention over the course of several weeks. Our intervention design also allows us to disentangle the effects of learning to read from typical brain development that occurs with age. The increased detectability of VWFA-2 in the intervention group and not the dyslexic control group is particularly notable, as it suggests that learning to read can uniquely stimulate the development of critical brain regions that may be underdeveloped in dyslexic readers. This finding is encouraging, as it demonstrates the brain's plasticity and its ability to adapt in response to a targeted educational environment.

In spite of significant changes in VWFA characteristics following the intervention, our findings suggest that the gap in VWFA size for dyslexic readers compared to typical readers remains, suggesting potential constraints on the extent of neural plasticity in this region. Typical readers continued to show greater text-evoked percent signal change in VWFA-1 compared to intervention participants. Additionally, the elevated object sensitivity observed in dyslexic readers persisted post-intervention, indicating that this patch of cortex continues to respond broadly to different visual categories with less specificity for words. While text selectivity increased for intervention participants in VWFA-2, it did not show a significant improvement in VWFA-1. These persistent differences raise important questions about the limitations of current interventions and the potential need for additional or alternative strategies.

Together, these findings offer a nuanced view of brain plasticity, where intervention can partially improve VWFA function but may not fully normalize it. This supports the notion that while targeted interventions can drive functional changes in the dyslexic brain, some neurostructural differences may be inherently stable traits that require prolonged or more intensive interventions. The dual findings of plasticity and stability imply that VWFA characteristics are influenced by both inherent, developmental factors and responsive, experience-driven changes.

This study is the first to closely compare intervention and control groups across multiple time points, delineating the trajectory of change within one year. Our findings of long lasting changes in the VWFA well after the completion of the intervention emphasize the potential for long-term enhancement through specific, targeted educational programs. The changes observed indicate that particular learning experiences can bring about measurable plasticity, while the stable traits reveal constraints on the plasticity of higher level visual cortex. Future research should build on these findings by conducting longitudinal analyses across diverse populations and extended timeframes, further investigating the neural mechanisms underlying these functional changes. These insights will be instrumental in understanding effective educational programs and designing interventions to support children with dyslexia.

In conclusion, we provide robust evidence that dyslexic readers exhibit significant reductions in VWFA size and specialization compared to typical readers, and that targeted interventions can partially mitigate these differences. However, the persistence of some disparities demonstrates a neural characteristic of dyslexia that remains despite behavioral evidence of remediated reading ability. This highlights the need for ongoing research to optimize intervention strategies and to explore the full potential of neuroplasticity in supporting literacy development in children with dyslexia. Further, this has broader implications to our understanding of learning induced plasticity in the human brain. The intervention design used here provides an unparalleled opportunity to examine the effects and limits of intensive learning in high-level visual cortex, and uncovers principles which may apply to other cognitive domains.

## Methods

### Participants

A total of 90 participants enrolled in the study. Three participants dropped out of the study before any usable fMRI data could be collected. Our final sample for analysis included 87 participants that had at least one usable run of the functional localizer at any given time point of the study. See Table 1 for a breakdown of age, gender, and scores by participant group for the final study sample.

All participants had normal or corrected-to-normal vision, no neurological or hearing issues, and were either monolingual English speakers or used English for at least 60% of their day, having learned the language before age three. The study protocols were approved by the Stanford School of Medicine Institutional Review Board, and informed assent from the children and written consent from their guardians were obtained prior to participation.

Participants were screened in a 45 minute virtual meeting. Participants were screened using the Wechsler Abbreviated Scale of Intelligence, Second Edition (WASI-II)^33^, ensuring scores above the 16th percentile for their age group. Participants who scored below a standard score of 85 on either the Woodcock-Johnson Basic Reading Skills (WJ BRS)^34^ or the Test of Word Reading Efficiency (TOWRE Index)^35^ were classified as “dyslexic” for the purposes of this study (either as an intervention or a control participant). Four participants were not available for a pre-study screening. All four participants had no parent reported reading difficulties and were classified as typical controls based on assessment data from their first study visit; all participants scored over 100 on TOWRE.

### Study Timeline

Participants completed several visits over a 13 month period. Participants completed a baseline visit before the start of the intervention period (or at the beginning of summer for control participants). Eight weeks later and after they finished the intervention, participants returned for their first follow-up visit. Two additional follow-up visits were completed approximately 6 months and 1 year after the start of the intervention. Intervention group participants also completed an additional pre-baseline visit that occurred one to two months before the baseline visit. See Supplementary Figure S3 for more detail. Behavioral assessments for each time point were collected no more than 2 weeks after the in-person scanning visit.

### Reading Intervention

Participants in the intervention group completed 160 hours of the Seeing Stars: Symbol Imagery for Fluency, Orthography, Sight Words, and Spelling program ^36^ - a curriculum from Lindamood-Bell that has been extensively studied and shown to improve reading ability in children with dyslexia ^28,37,38^. The intervention was delivered for 4 hours a day, 5 days a week, over 8 weeks of the participants' summer vacation from school. Due to the COVID-19 pandemic, the sessions were conducted remotely via Zoom. All sessions were led by certified Lindamood-Bell instructors. The individualized, multisensory curriculum focused on phonological and orthographic skills, progressing from letters to connected text. Children were guided through activities like air-writing and visualizing letter-sound connections to build literacy foundations. The program emphasized decoding, spelling, fluency, and comprehension. All participants received the intervention at no cost. Dyslexic control group participants were offered free access to the same intervention the summer after their participation in the study.

### Behavioral Assessments

Each visit included a thorough assessment of reading and cognitive skills by trained researchers at Stanford University. The repeated tests at each visit included the Woodcock-Johnson IV (WJ) and Test of Word Reading Efficiency-2 (TOWRE). WJ subtests were used to create Basic Reading Skills (BRS; Letter-Word Identification - LWID, Word Attack - WA) and Reading Fluency (RF; Oral Reading - OR, Sentence Reading Fluency - SRF) composite scores ^34^. The TOWRE tests for Sight-Word Efficiency (SWE) and Phonemic Decoding Efficiency (PDE) were used to calculate the TOWRE Index ^35^. Additionally, the WJ Math Facts Fluency subtest was administered. Alternative test forms were used across visits to ensure reliability and mitigate practice effects. All assessments, except for SRF and MFF, were administered remotely via video call to reduce face-to-face contact between participants and researchers as the study began during the COVID-19 pandemic. Assessment video call sessions were video and audio recorded and all assessments were independently scored by 2 or more trained researchers.

### fMRI Experimental Design

The functional localizer experiment used in the study was an adaptation of White and colleagues’ (2023) functional localizer experiment. For this experiment, participants viewed 5 main categories of visual stimuli: text, pseudo fonts, objects, faces, and limbs (Figure 1). Each category was composed of more specific subcategories. Text contained high and low frequency words, pseudo words, and consonant strings; pseudo fonts was composed of false fonts designed to contain similar visual features to the Sloan and Courier fonts; objects contained images of random objects like fruits, construction machinery, instruments, and sunglasses; faces contained images of male and female faces facing forward and to either side; and limbs contained images of disembodied hands, arms, feet, and legs. Stimuli were presented in groups of three (all from the same subcategory) on a single frame with a large image on either side of fixation and a smaller image at the center of the screen under the fixation dot (See Figure 1). Size of stimuli were set to balance the visual acuity and processing advantage of fovea and periphery such that they would use comparable amounts of cortical territory.

A single trial consisted of 4 frames of triplets of the same category. Each frame was presented for 800 ms, followed by a 200 ms blank fixation screen. Each subcategory was displayed 5 times during a run and category order was randomly assigned. Additionally, 5 blocks of blank screen trials were randomly dispersed during a run and every run began and ended with a 5 second blank screen.

During each run of the experiment, participants were asked to perform a task; a one-back image repetition task or a fixation color change task. During the one-back task, participants were instructed to press a button every time the image on the screen repeated. During the fixation task, participants were instructed to press a button every time the fixation dot changed colors. Participants used the index finger of their dominant hand to respond. Fixation and one-back targets occurred randomly during 33% of trials in every run, regardless of the instructions provided to the participant.

Participants were monitored via a web camera to ensure they were awake and attending to the task. If a participant fell asleep during the run, the run was stopped and excluded from analyses. Participants completed 4 runs of the experiment (2 of each task) at each visit of the study.

### MRI Acquisition

Participants were scanned using a General Electric Sigma MR750 3T scanner at Stanford University’s Center for Cognitive and Neurobiological Imaging (CNI). Before the main scanning session, participants attended an introductory session where they practiced a brief version of the experiment in a mock scanner. This session helped them get accustomed to the scanner noises, tasks, and response box. They also practiced staying still with the aid of the MoTrak Head Motion Tracking System (RRID:SCR_009607), which provided feedback on their motion.

Functional runs were collected using a gradient echo EPI sequence with a multiband factor of 3, ensuring whole-brain coverage across 51 slices. The acquisition parameters included a TR of 1.19s, a TE of 30ms, and a flip angle of 62, resulting in a spatial resolution of 2.4 mm^3^ isotropic voxels. Each run consisted of 232 frames and lasted 4 minutes and 36 seconds. Additionally, a high-resolution T1-weighted anatomical scan was acquired with a spatial resolution of 0.9 mm3 isotropic voxels.

### MR Proprocessing

Functional data preprocessing was carried out using fMRIPrep version 23.1.3 ^39^, which is built upon Nipype version 1.8.6 ^40,41^.

#### Anatomical Data Preprocessing

The T1-weighted (T1w) image was first reoriented to align with the AC-PC axis using ANTSpy version 0.4.2 ^42^ and served as the anatomical reference throughout the preprocessing workflow. The T1w image was then skull-stripped with Synthstrip ^43^ and processed through FreeSurfer’s recon-all pipeline (version 7.3.2; ^44^) using the Synthseg robust algorithm ^45^ for segmentation and surface reconstruction. All surfaces underwent visual inspection, with manual edits made where necessary. The resulting FreeSurfer derivatives were subsequently utilized by the fMRIPrep pipeline. Segmentation of brain tissue into cerebrospinal fluid (CSF), white matter (WM), and gray matter (GM) was performed on the skull-stripped T1w image using FAST (FSL, RRID; ^46^). Spatial normalization to a standard space (MNI152NLin2009cAsym) was achieved through nonlinear registration with antsRegistration (ANTs; Tustison et al.), using skull-stripped versions of both the T1w reference and the T1w template.

#### Functional Data Preprocessing

For each BOLD run, a reference volume was created using a custom method implemented in fMRIPrep. Before any spatiotemporal filtering, head-motion parameters relative to the BOLD reference (including transformation matrices and six rotation and translation parameters) were estimated using mcflirt (FSL; ^47^). A B0 nonuniformity map was estimated from two echo-planar imaging (EPI) references using topup ^48^, and the estimated fieldmap was rigidly aligned to the target EPI reference run. The field coefficients were then applied to the reference EPI using the appropriate transformation. Slice-time correction was performed on the BOLD runs to align all slices to the middle slice using 3dTshift from AFNI (^49^; RRID). The BOLD reference was co-registered to the T1w reference using bbregister (FreeSurfer), which implements boundary-based registration ^50^. Framewise motion parameters and BOLD signal within the white matter (WM) and corticospinal fluid (CSF) were calculated and later used as confound regressors in the BOLD response estimation. The BOLD time-series were resampled into standard space, resulting in preprocessed BOLD runs in MNI152NLin2009cAsym space, and were also resampled onto the fsnative and fsaverage FreeSurfer surfaces. All resampling steps were executed with a single interpolation process, combining all necessary transformations (e.g., head-motion correction, susceptibility distortion correction, and co-registration to anatomical and output spaces). Volumetric resampling was conducted using antsApplyTransforms (ANTs) with Lanczos interpolation to reduce smoothing effects, while surface resampling was performed using mri_vol2surf (FreeSurfer).

#### BOLD Response Estimation

We used mriqc (version 22.0.1;^51^) to assess data quality and exclude noisy runs. Runs were excluded from analysis if the mean framewise displacement (FD) was 0.5 mm or larger, or if more than 30% of frames had an FD greater than 0.5 mm. Additionally, runs where participants failed to keep their eyes open were also excluded. BOLD response estimation was carried out by fitting a general linear model (GLM) to the BOLD time-series in each participant’s native surface using Nilearn (version 0.5.0). The design matrix for the GLM included signals from the white matter and CSF, along with their first derivatives, as well as the six motion parameters. First- and second-order polynomial drift terms were also included. The estimated BOLD responses (beta weights) are reported in units of percent signal change, reflecting the change in response relative to blank trials where participants viewed a blank fixation screen.

### Region of Interest Definition

Regions of interest (ROIs) were manually drawn on the native surface of every participant using the Freeview viewer of FreeSurfer Suite^52^. To determine exact boundaries of these ROIs, we calculated statistical maps that compared the estimated BOLD response of a target category (i.e. text) to the other four remaining categories (i.e. pseudo fonts, faces, limbs, objects). Contrasts were weighted by the number of sub categories to ensure balance across the varying number of blocks per stimulus category. Maps were thresholded at t-value > 3 and defined with the following anatomical constraints: All ROIs were defined within the bounds of the ventral occipitotemporal cortex (VOTC); posterior of the anterior tip of the occipitotemporal sulcus (OTS), lateral to the collateral sulcus, anterior to the posterior transverse collateral sulcus, and medial to and inclusive of the OTS. VWFA clusters were defined using any vertices that met threshold value which fell on the left occipitotemporal sulcus and lateral portion of the fusiform gyrus (any patch of cortex lateral to the mid-fusiform sulcus). FFA clusters were defined using any vertices that met threshold value which fell on the fusiform gyrus and mid fusiform sulcus. Continuous clusters of activation that extended further medially or laterally that the described anatomy were bounded by the borders of the described anatomy.

The more posterior VWFA-1 and FFA-1 were discerned from the more anterior VWFA-2^53^ and FFA-2 using the boundaries of the Fusiform Gyrus cytoarchitectonic FG2 & FG4 ROIs from Rosenke et al. ^54^. These template ROIs were projected from average space to the native space of every participant. VWFA-1 and FFA-1 were roughly extended to the anterior boundary FG2 while VWFA-2 and FFA-2 extended from the posterior boundary of FG4 until the anterior tip of the fusiform gyrus. Continuous patches of activation were split at natural saddles in activation that fell closest to these anterior and posterior boundaries. Medial and lateral boundaries of native anatomy were strictly adhered to.

Two sets of ROIs were drawn with these criteria. The first set of ROIs, on which the majority of analysis was conducted, used data from every available run for each participant across all time points to create statistical maps. These “combined session” ROIs were used for all analyses involving category selectivity. With this approach, we were able to define VWFA-1 in 73 participants and VWFA-2 in 66 participants out of the 87 total participants. A second set of ROIs was drawn for each time point of the study using statistical maps that were created from all available runs (Supplementary Figure S4) per study time point. These “separate session” ROIs were used for analyses of VWFA emergence and size. See Supplementary (Figure S5 and Figure S6) for images of all ROIs drawn for this study.

### Statistical Analysis

#### Response Estimation

Activation maps for neural responses to the visual localizer were created by fitting general linear models convolving the SPM hemodynamic response function to stimulus onsets using the nilearn package in python^55,56^. This approach was used to create two different types of heat maps. First, statistical maps displaying response preference for one visual category compared to others were created to define functional ROIs. For defining VWFAs, this was done by contrasting the response to text (high frequency and low frequency words, consonant strings, and pseudo words) to response to all other visual categories (faces, false fonts, objects, and limbs). A similar process was done for defining FFAs with contrasts of faces to other visual categories. Resulting maps were reported as *t*-statistics for every vertex on the cortical surface. For analyzing response profiles within ROIs, heat maps were created for each of the visual categories with values of percent signal change from baseline. These values were averaged across all vertices in each ROI.

#### Linear Mixed-Effects Modeling

We used the *lme4* package in R^57^ to run linear mixed effects models (LMEs) to investigate differences in baseline percent signal change, and all longitudinal changes in assessment scores, size, signal, and selectivity. All LMEs that utilized neuroimaging data also included covariates of participant age at baseline, along with the mean framewise displacement and the number of usable runs per session of data collection. Every time an LME included a group level parameter and interaction, the intervention group was set af the reference category. In baseline analyses, the dyslexic group was set as the reference category. Similarly, every time an LME included an analysis of percent signal change, response to text was set as the reference category.

## Supplementary Material

Supplement 1

## Figures and Tables

**Figure 5 ∣:**
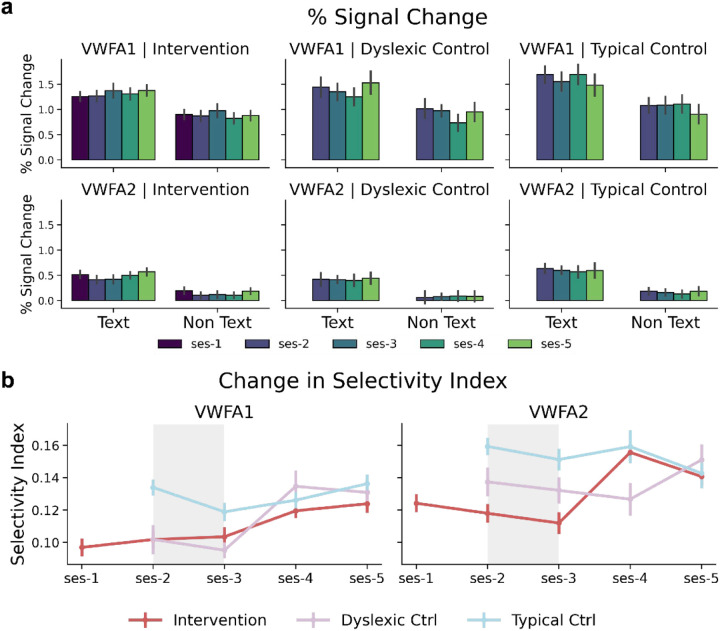
VWFA Does Not Show Intervention Driven Increases of Text Sensitivity **a**, BOLD response (in units of percent signal change) to text (left) and non-text (right) categories, at each time point, within VWFA-1 (top) and VWFA-2 (bottom). Error bars represent standard error. **b**, Change in selectivity index across each time point in VWFA-1 (left) and VWFA-2 (right). Error bars represent the between-participant standard error.

**Table 1 ∣:** LME results for the relationship between ROI size and assessment state and trait WJ BRS: Woodcock-Johnson Basic Reading Skills; WJ RF: Woodcock-Johnson Reading Fluency; TOWRE: Test of Word Reading Efficiency; WJ MFF: Woodcock-Johnson Math Facts Fluency; Dys Ctrl: Dyslexic Control; Typ Ctrl: Typical Control; FD: Framewise Displacement; Trait: within-participant average over time; State: within-participant change relative to trait.

Log Size − Age + Movement + RunNums + Reading Trait + Reading State*Subgroup (1 ∣ Participant)												
	VWFA1			VWFA2			FFA1			FFA2		
	β	t	p	β	t	p	β	t	p	β	t	p
WJ BRS												
Intercept	−5.5207	−2.9833	**0.0035**	−7.5591	−3.4354	**0.0008**	2.6632	2.4048	**0.0182**	2.9672	2.2952	**0.0241**
WJ BRS Trait	0.0482	2.8844	**0.0049**	0.0828	4.0769	**0.0001**	0.0115	1.1476	0.2549	−0.0124	−1.0334	0.3047
Group: Dys Ctrl	−0.6436	−1.6208	0.1089	−0.1535	−0.3155	0.7532	−0.1147	−0.4800	0.6328	−0.1807	−0.6251	0.5339
Group: Typ Ctrl	0.3212	0.5972	0.5520	−0.2380	−0.3616	0.7186	−0.4568	−1.4111	0.1627	−0.4454	−1.1403	0.2579
WJ BRS State	0.0530	3.4267	**0.0007**	0.0696	4.6615	**5.18E-06**	−0.0048	−0.5368	0.5919	−0.0051	−0.6433	0.5206
Age	0.3823	3.3860	**0.0011**	0.2058	1.4885	0.1403	0.0431	0.6335	0.5285	0.1649	2.0080	**0.0484**
Movement (Mean FD)	−1.0562	−1.2804	0.2013	−0.4189	−0.5044	0.6143	−1.1497	−2.3929	**0.0173**	−0.2153	−0.4792	0.6322
# of Runs	0.3037	2.2939	**0.0225**	0.4172	3.1957	**0.0016**	0.5815	7.5710	**5.52E-13**	0.6168	8.7970	**2.01E-16**
Group: Dys Ctrl * WJ BRS State	−0.0456	−1.1906	0.2350	−0.0563	−1.5226	0.1291	−0.0145	−0.6571	0.5118	−0.0118	−0.5989	0.5499
Group: Typ Ctrl * WJ BRS State	−0.0623	−1.6165	0.1073	−0.0643	−1.7254	0.0857	0.0090	0.4046	0.6861	0.0032	0.1600	0.8730
WJ RF												
Intercept	−2.9943	−1.9351	0.0555	−4.4244	−2.4385	**0.0164**	4.1797	5.0130	**2.13E-06**	3.8182	3.9354	**0.0002**
WJ RF Trait	0.0287	2.0701	**0.0414**	0.0663	3.9904	**0.0001**	0.0006	0.0755	0.9400	−0.0212	−2.3889	**0.0193**
Group: Dys Ctrl	−0.9046	−2.3108	**0.0233**	−0.5900	−1.2483	0.2153	−0.1500	−0.7195	0.4740	−0.0887	−0.3523	0.7256
Group: Typ Ctrl	0.7769	1.4406	0.1536	0.0164	0.0251	0.9800	−0.0290	−0.1012	0.9197	0.0158	0.0454	0.9639
WJ RF State	0.0854	4.8731	**1.98E-06**	0.1106	6.6221	**2.24E-10**	−0.0139	−1.3813	0.1685	−0.0131	−1.4306	0.1539
Age	0.3396	2.8937	**0.0049**	0.1144	0.8058	0.4226	−0.0082	−0.1318	0.8955	0.1365	1.8041	0.0753
Movement (Mean FD)	−0.9801	−1.2099	0.2272	−0.2738	−0.3409	0.7334	−1.3729	−2.9939	**0.0030**	−0.3869	−0.8802	0.3795
# of Runs	0.2280	1.7652	0.0786	0.3030	2.4253	**0.0160**	0.5729	7.7603	**1.59E-13**	0.6313	9.2057	**1.16E-17**
Group: Dys Ctrl * WJ RF State	−0.0428	−1.0529	0.2935	−0.0937	−2.4232	**0.0161**	−0.0083	−0.3557	0.7224	−0.0032	−0.1529	0.8786
Group: Typ Ctrl * WJ RF State	−0.0865	−2.3641	**0.0189**	−0.1381	−3.9720	**0.0001**	−0.0199	−0.9461	0.3451	−0.0086	−0.4489	0.6539
TOWRE												
Intercept	−4.9536	−3.0371	**0.0030**	−7.2896	−4.0355	**0.0001**	2.6336	2.7041	**0.0081**	2.2712	1.9787	0.0509
TOWRE Trait	0.0561	3.1840	**0.0020**	0.1165	5.8508	**7.82E-08**	0.0204	1.9301	0.0575	−0.0044	−0.3450	0.7310
Group: Dys Ctrl	−0.4456	−1.1022	0.2735	0.2962	0.6452	0.5205	−0.0636	−0.2619	0.7942	−0.1571	−0.5283	0.5989
Group: Typ Ctrl	−0.0148	−0.0245	0.9805	−1.3116	−1.9189	0.0582	−0.7285	−2.0099	**0.0482**	−0.6418	−1.4549	0.1498
TOWRE State	0.0389	2.3183	**0.0213**	0.0575	3.6395	**0.0003**	−0.0023	−0.2337	0.8154	0.0072	0.8366	0.4037
Age	0.2905	2.4598	**0.0160**	−0.0079	−0.0591	0.9530	0.0060	0.0845	0.9329	0.1694	1.9528	0.0547
Movement (Mean FD)	−0.8446	−1.0048	0.3158	−0.2859	−0.3487	0.7275	−1.2338	−2.5183	**0.0123**	−0.1396	−0.3052	0.7604
# of Runs	0.3253	2.4291	**0.0158**	0.3993	3.1152	**0.0020**	0.5397	6.9437	**2.78E-11**	0.5939	8.4293	**2.67E-15**
Group: Dys Ctrl * TOWRE State	−0.0608	−1.4229	0.1561	−0.0403	−1.0020	0.3173	0.0221	0.8951	0.3717	0.0147	0.6748	0.5005
Group: Typ Ctrl * TOWRE State	−0.0335	−0.8262	0.4095	−0.0321	−0.8432	0.3999	0.0031	0.1317	0.8954	−0.0031	−0.1523	0.8791
WJ MFF												
Intercept	−1.5049	−0.9235	0.3578	−2.3901	−1.1908	0.2366	3.2400	3.4361	**0.0009**	2.5121	2.2845	**0.0248**
WJ MFF Trait	−0.0020	−0.1789	0.8585	0.0179	1.2293	0.2225	0.0060	0.9020	0.3706	−0.0055	−0.6816	0.4977
Group: Dys Ctrl	−0.8740	−2.1253	**0.0365**	−0.4604	−0.8861	0.3781	−0.1644	−0.6881	0.4938	−0.1623	−0.5659	0.5732
Group: Typ Ctrl	1.5284	3.5927	**0.0006**	1.4730	2.7373	**0.0076**	−0.2699	−1.0917	0.2790	−0.6437	−2.1660	**0.0336**
WJ MFF State	0.0165	0.8306	0.4070	0.0098	0.4999	0.6176	0.0138	1.2426	0.2153	0.0097	0.9852	0.3256
Age	0.4144	3.5112	**0.0007**	0.2613	1.7552	0.0828	0.0467	0.6808	0.4983	0.1539	1.8738	0.0649
Movement (Mean FD)	−1.4808	−1.7651	0.0785	−0.9912	−1.1451	0.2531	−1.2442	−2.6279	**0.0090**	−0.1804	−0.4091	0.6828
# of Runs	0.3029	2.2685	**0.0240**	0.3919	2.9110	**0.0039**	0.5490	7.3133	**2.91E-12**	0.6058	8.8955	**1.05E-16**
Group: Dys Ctrl * WJ MFF State	−0.0300	−0.9013	0.3683	−0.0146	−0.4446	0.6570	−0.0375	−2.0128	**0.0453**	−0.0394	−2.3814	**0.0181**
Group: Typ Ctrl * WJ MFF State	0.0175	0.4238	0.6721	−0.0287	−0.7020	0.4834	−0.0448	−1.9350	0.0543	−0.0241	−1.1711	0.2428

**Table 2 ∣:** Participant Information and Behavioral Results at First Visit Demographic information for participants divided by study group. WJ BRS: Woodcock-Johnson Basic Reading Skills; WJ RF: Woodcock-Johnson Reading Fluency; TOWRE: Test of Word Reading Efficiency; WJ MFF: Woodcock-Johnson Math Facts Fluency.

	Intervention Group	Dyslexic Control Group	Typical Control Group
Total Count	44	19	24
Gender (f/m)	20 / 23	9 / 10	13 / 11
Mean age ± std	9.8 ± 1.1	10.2 ± 1.8	9.9 ± 1.4
Behavioral Assessments			
mean score ± std			
WJ BRS	80.4 ± 11.4	81.5 ± 11.4	110.1 ± 8.5
WJ RF	74.0 ± 13.8	76.5 ± 12.0	103.7 ± 9.9
TOWRE Index	72.2 ± 9.9	71.2 ± 9.8	106.2 ± 10.3
WJ MFF	82.0 ± 15.1	79.3 ± 9.0	101.4 ± 13.0
Functional Localizer Task Accuracy			
mean ± std			
hit rate	0.592 ± 0.205	0.562 ± 0.203	0.649 ±0.211
*d'*	2.555 ± 0.660	2.451 ± 0.617	2.736 ± 0.718
