# Supplementary material for: Small or absent Visual Word Form Area is a trait of dyslexia
Source: bioRxiv. 2025 Jan 15:2025.01.14.632854. Preprint. [Version 1] doi: 10.1101/2025.01.14.632854 (PMC11761755; doi:10.1101/2025.01.14.632854)
Supplement: Supplement 1 [file media-1.pdf]

Small or absent Visual Word Form Area is a trait of dyslexia: Supplementary Material

Jamie L. Mitchell[1,2], Maya Yablonski[1,3], Hannah L. Stone[1,4], Mia Fuentes-Jimenez[1], Megumi E. Takada[1], Kenny A. Tang[5], Jasmine E. Tran[1,6], Clementine Chou[1], Jason D. Yeatman[1,2,3]

[1]Graduate School of Education, Stanford University, Stanford, CA, USA.
[2]Department of Psychology, Stanford University, Stanford, CA, USA.
[3]Division of Developmental-Behavioral Pediatrics, Department of Pediatrics, Stanford University School of Medicine, Stanford,California, USA.
[4]Department of Psychological & Brain Sciences, University of California, Santa Baraba, CA, USA.
[5]Department of Special Education, Peabody College of Education and Human Development, Vanderbilt University, Nashville, TN, USA.
[6]School of Education, University of California, Irvine, CA, USA.

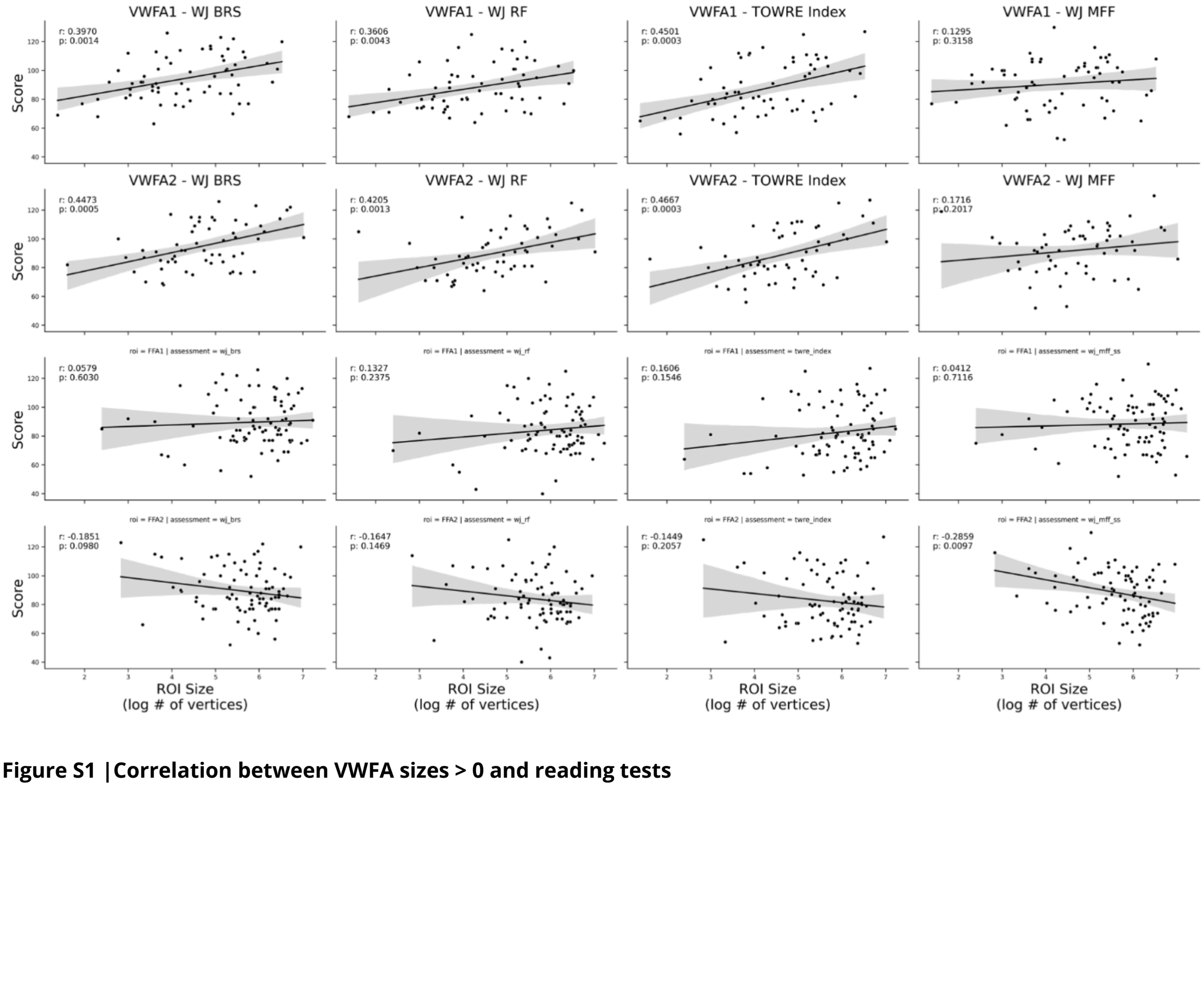

Figure S1 |Correlation between VWFA sizes > 0 and reading tests

Mean % Signal Change ~ age + movement + RunNums + category*subgroup + (1 | sub)												
	VWFA1			VWFA2			FFA1			FFA2		
	β	t	p	β	t	p	β	t	p	β	t	p
Intercept (Dyslexic Readers | Text)	1.1882	1.2659	0.2101	0.1140	0.2007	0.8416	0.3216	0.4603	0.6466	1.2546	3.1099	**0.0026**
Category: Pseudo Fonts	-0.2016	-3.3832	**0.0008**	-0.2289	-6.4851	**4.77E-10**	-0.0307	-0.6470	0.5181	-0.0570	-1.7372	0.0833
Category: Objects	-0.2572	-4.3159	**2.23E-05**	-0.2972	-8.4200	**3.05E-15**	1.0091	21.2923	**1.55E-63**	0.4114	12.5388	**1.51E-29**
Category: Faces	-0.8450	-14.1819	**1.49E-34**	-0.4718	-13.3661	**4.72E-31**	1.1690	24.6682	**2.30E-76**	0.6836	20.8343	**2.85E-61**
Category: Limbs	-0.3280	-5.5054	**8.52E-08**	-0.2807	-7.9526	**6.48E-14**	0.9042	19.0806	**6.24E-55**	0.5312	16.1902	**2.36E-43**
Group: Typical Readers	0.3687	2.0843	**0.0401**	0.2060	1.9457	0.0553	0.1765	1.2083	0.2296	0.0038	0.0441	0.9649
Age	0.1255	2.1352	**0.0365**	0.0382	0.9949	0.3238	0.0710	1.5039	0.1366	-0.0188	-0.7037	0.4837
movement (Mean FD)	-0.9050	-1.2854	0.2032	-0.3770	-0.9241	0.3592	-0.4517	-0.8058	0.4228	-1.0962	-3.4264	**0.0010**
# of Good Runs	-0.2610	-1.7583	0.0834	0.0037	0.0440	0.9650	-0.0257	-0.2487	0.8043	-0.1286	-2.0757	**0.0413**
Category: Pseudo Fonts * Group: Typical Readers	-0.1813	-1.7443	0.0822	-0.1292	-2.0974	**0.0370**	-0.0922	-1.0459	0.2964	-0.1064	-1.7272	0.0851
Category: Objects * Group: Typical Readers	-0.3750	-3.6076	**0.0004**	-0.1993	-3.2351	**0.0014**	-0.2818	-3.1981	**0.0015**	-0.1898	-3.0824	**0.0022**
Category: Faces * Group: Typical Readers	-0.0662	-0.6367	0.5249	-0.1412	-2.2916	**0.0228**	-0.1029	-1.1676	0.2438	-0.1108	-1.7999	0.0728
Category: Limbs * Group: Typical Readers	-0.1927	-1.8543	0.0648	-0.0542	-0.8799	0.3797	-0.1022	-1.1596	0.2471	-0.0814	-1.3225	0.1870

Table S1 | Cross-sectional LME results for the relationship between reading ability group and response to different visual category

Text Selectivity Index & Score Correlations								
	VWFA1		VWFA2		FFA1		FFA2	
Assessment	r	p	r	p	r	p	r	p
WJ BRS	0.4041	**0.0006**	0.3673	**0.0033**	0.2623	**0.0180**	0.3353	**0.0024**
WJ RF	0.3750	**0.0016**	0.0377	**0.0027**	0.1525	0.1798	0.3998	**0.0003**
TOWRE Index	0.3450	**0.0046**	0.3837	**0.0027**	0.2365	**0.0371**	0.3522	**0.0017**
WJ MFF	0.1458	0.2321	0.1226	0.3423	0.0807	0.4741	0.2560	**0.0219**

Table S2 | Pearson R results for the correlation between reading assessment score and text selectivity index

Score ~ Time + (1 | Participant)	Intervention Group			Dyslexic Control Group			Typical Control Group		
	β	t	p	β	t	p	β	t	p
WJ BRS									
Intercept	82.5771	57.7649	**2.14E-45**	80.1853	32.2661	**1.28E-18**	110.0395	62.6679	**1.20E-31**
Time (Days from ses2)	0.0205	6.9447	**7.98E-11**	-0.0044	-0.8085	0.4230	-0.0059	-1.0887	0.2814
WJ RF									
Intercept	75.4453	40.1198	**1.54E-36**	76.8346	29.6078	**3.26E-18**	105.1071	47.0453	**1.08E-25**
Time (Days from ses2)	0.0188	7.5371	**3.08E-12**	0.0087	1.7201	0.0921	0.0209	4.4414	**4.92E-05**
TOWRE Index									
Intercept	73.3317	50.5512	**8.93E-42**	69.7882	36.5120	**1.54E-21**	103.8160	52.8638	**4.99E-27**
Time (Days from ses2)	0.0252	10.3977	**8.36E-20**	0.0035	0.6668	0.5082	-0.0038	-0.6883	0.4947
WJ MFF									
Intercept	82.7249	35.0205	**6.01E-34**	78.6398	35.1971	**1.60E-22**	100.0639	37.3751	**5.33E-24**
Time (Days from ses2)	0.0066	2.5160	**0.0128**	0.0005	0.0665	0.9473	0.0093	1.8030	0.0775

Score ~ Time + (1 | Participant)	WJ BRS			WJ RF			TOWRE Index			WJ MFF		
	β	t	p	β	t	p	β	t	p	β	t	p
Intercept | Intervention	82.5770	58.0560	**0.0000**	75.4463	42.4869	**0.0000**	73.3301	52.7782	**0.0000**	82.7292	39.6468	**0.0000**
Time (Days from ses2)	0.0205	7.2122	**0.0000**	0.0188	7.7172	**0.0000**	0.0252	10.2857	**0.0000**	0.0067	2.3922	**0.0175**
Group: Dys Ctrl	-2.4328	-0.9272	0.3561	1.3877	0.4307	0.6677	-3.5303	-1.3803	0.1707	-4.1040	-1.0841	0.2811
Group: Typ Ctrl	27.4561	11.2068	**0.0000**	29.6602	9.7701	**0.0000**	30.4745	12.6676	**0.0000**	17.3440	4.9244	**0.0000**
Group: Dys Ctrl * Time	-0.0248	-3.8186	**0.0002**	-0.0101	-1.8140	0.0708	-0.0218	-3.7975	**0.0002**	-0.0061	-0.9534	0.3412
Group: Typ Ctrl * Time	-0.0263	-3.9768	**0.0001**	0.0021	0.3757	0.7074	-0.0290	-4.9708	**0.0000**	0.0025	0.3853	0.7004

Table S3 | LME results for change in assessment score over time for each of the three participant groups separately (top) and with group interactions (bottom)

Log Size ~ age + movement + RunNums + Time*Group + (1 | Participant)	VWFA1			VWFA2			FFA1			FFA2		
	β	t	p	β	t	p	β	t	p	β	t	p
Intercept	-2.2512	-1.7183	0.0883	-1.5351	-0.9497	0.3444	3.5769	4.6430	**1.06E-05**	1.8552	2.1009	**0.0383**
Time (Days from ses2)	0.0032	5.2164	**3.87E-07**	0.0032	5.2260	**3.73E-07**	0.0002	0.4275	0.6694	0.0002	0.5324	0.5949
Group: Dys Ctrl	-0.9232	-2.1456	**0.0340**	-0.4931	-0.9196	0.3600	-0.4005	-1.5837	0.1167	-0.3693	-1.2588	0.2114
Group: Typ Ctrl	1.6957	4.2757	**3.97E-05**	2.3656	4.7894	**5.78E-06**	-0.0251	-0.1078	0.9144	-0.6636	-2.4556	**0.0160**
Age	0.4347	3.7434	**0.0003**	0.2795	1.8799	0.0635	0.0564	0.8277	0.4108	0.1667	2.0431	**0.0446**
Movement (Mean FD)	-0.7711	-0.9661	0.3347	-0.3101	-0.3750	0.7079	-1.1433	-2.4213	**0.0160**	-0.0131	-0.0299	0.9762
Num Good Runs	0.2608	2.0804	**0.0384**	0.3762	2.9681	**0.0033**	0.5578	7.5112	**8.44E-13**	0.6103	9.0917	**2.68E-17**
Time * Group: Dys Ctrl	0.0002	0.1165	0.9074	-0.0008	-0.5961	0.5517	0.0018	2.2100	**0.0281**	0.0021	2.9054	**0.0040**
Time * Group: Typ Ctrl	-0.0014	-1.0420	0.2984	-0.0049	-3.6277	**0.0003**	-0.0014	-1.7015	0.0901	-0.0008	-1.1426	0.2543

Table S4 | LME results for change in ROI size over time

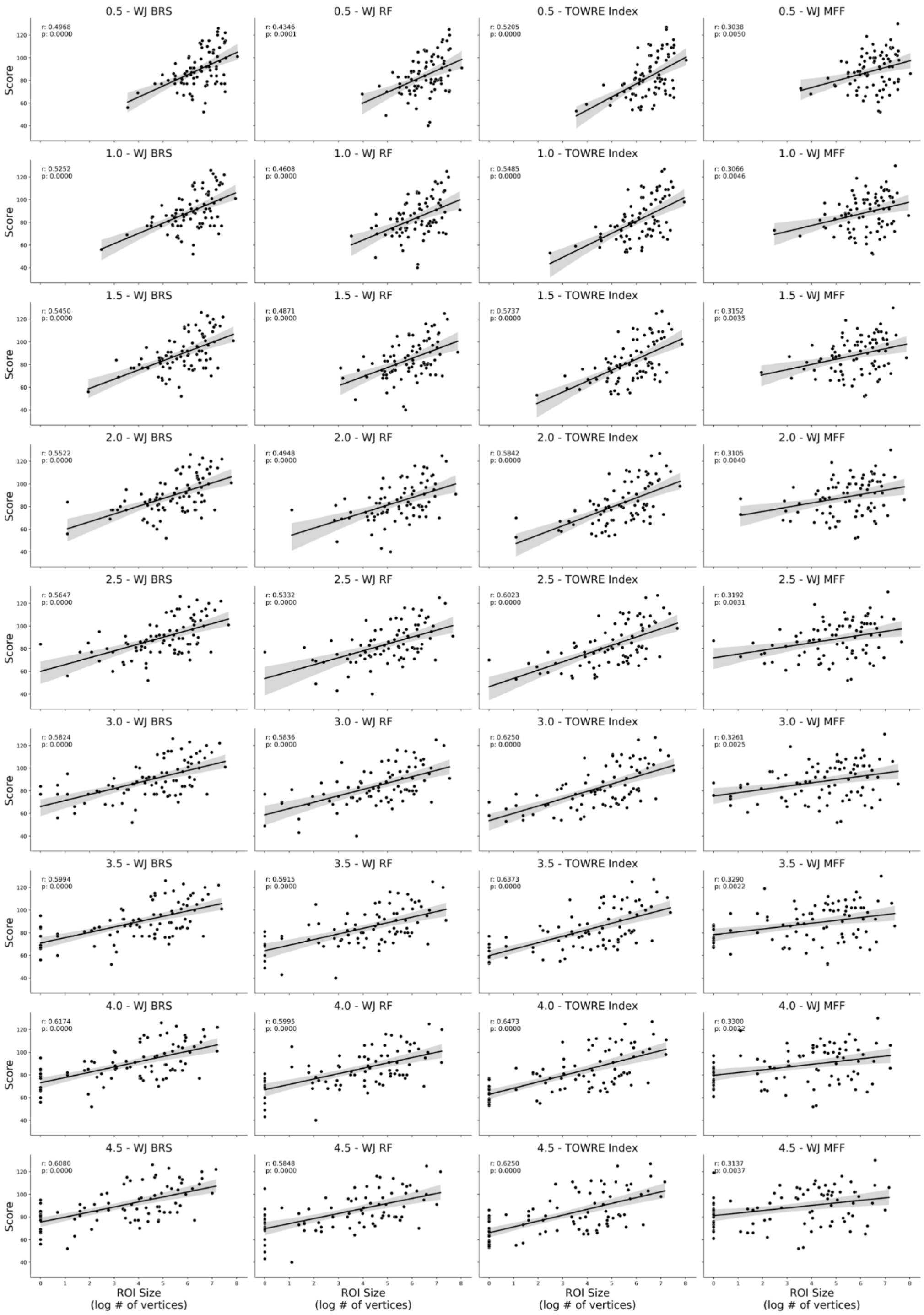

Figure S2 | Correlations between VWFA size and assessment across thresholds
Thresholds range from *t* = 0.5 to 4.5 in increments of 0.5.

PSC ~ age + Movement + Num Good Runs + Time * Category * Sub Group + (1 | Participant)												
	VWFA1			VWFA2			FFA1			FFA2		
	**β**	**t**	**p**	**β**	**t**	**p**	**β**	**t**	**p**	**β**	**t**	**p**
Intercept (Intervention | Text)	0.2052	0.3842	0.7018	0.3922	1.2187	0.2267	0.5529	1.7155	0.0891	0.3242	1.8951	0.0609
Age	0.1030	2.0540	**0.0437**	0.0251	0.8302	0.4095	0.0344	1.1493	0.2536	0.0078	0.4889	0.6262
Movement (Mean FD)	-0.1514	-0.8213	0.4117	-0.1029	-0.8297	0.4070	-0.3425	-2.3249	**0.0202**	-0.1970	-2.5116	**0.0121**
# of Good Runs	0.0070	0.2390	0.8112	-0.0350	-1.9176	0.0556	-0.0400	-1.7403	0.0821	-0.0212	-1.7278	0.0843
Time (days from ses2)	0.0002	1.3635	0.1731	0.0003	2.6366	**0.0086**	0.0000	0.1757	0.8605	0.0000	-0.3884	0.6978
Category: Not Text	-0.3782	-8.8514	**5.86E-18**	-0.3377	-11.2193	**7.18E-27**	0.8498	22.6532	**1.07E-94**	0.4324	21.5746	**3.06E-87**
Group: Dys Ctrl	0.0888	0.4552	0.6501	-0.0626	-0.5449	0.5873	-0.0348	-0.3037	0.7618	-0.0327	-0.5371	0.5922
Group: Typ Ctrl	0.3809	2.3868	**0.0192**	0.0878	0.9409	0.3494	0.1818	1.7368	0.0849	0.0609	1.0912	0.2774
Time * Category: Not Text	-0.0003	-1.3670	0.1720	-0.0002	-1.3647	0.1728	0.0000	0.0068	0.9946	0.0000	0.1393	0.8892
Time * Group: Dys Ctrl	0.0001	0.3379	0.7355	-0.0001	-0.4332	0.6650	-0.0001	-0.3995	0.6896	0.0001	0.6775	0.4982
Time * Group: Typ Ctrl	-0.0005	-1.5050	0.1327	-0.0002	-1.0500	0.2941	-0.0003	-0.8308	0.4063	0.0001	0.6099	0.5420
Category: Not Text * Group: Dys Ctrl	-0.0236	-0.2194	0.8264	0.0038	0.0554	0.9558	-0.0867	-1.0519	0.2931	-0.0430	-0.9667	0.3339
Category: Not Text * Group: Typ Ctrl	-0.1830	-2.2562	**0.0243**	-0.0883	-1.5163	0.1299	-0.1154	-1.5179	0.1293	-0.0825	-2.0214	**0.0435**
Time * Category: Not Text * Group: Dys Ctrl	-0.0001	-0.0972	0.9226	0.0003	0.8692	0.3851	0.0003	0.7867	0.4316	0.0001	0.4877	0.6259
Time * Category: Not Text * Group: Typ Ctrl	0.0003	0.6163	0.5379	0.0004	1.1980	0.2314	-0.0003	-0.6607	0.5089	-0.0002	-1.0173	0.3092

Table S5 | LME results for change response magnitude to visual stimuli over time

Selectivity Index ~ age + Movement + Num Good Runs + Time * Sub Group + (1 | sub)												
	VWFA1			VWFA2			FFA1			FFA2		
	**β**	**t**	**p**	**β**	**t**	**p**	**β**	**t**	**p**	**β**	**t**	**p**
Intercept (Intervention)	0.0554	0.9048	0.3672	0.1006	1.6412	0.1035	-0.1624	-2.7763	**0.0064**	-0.1210	-2.4889	**0.0144**
Age	-0.0037	-0.7514	0.4548	-0.0005	-0.1108	0.9121	0.0016	0.3050	0.7611	0.0009	0.2085	0.8354
Movement (Mean FD)	0.0628	1.5259	0.1283	0.0055	0.1264	0.8995	-0.0867	-2.5698	**0.0107**	-0.0422	-1.8228	0.0695
# of Good Runs	0.0208	2.8645	**0.0045**	0.0089	1.2209	0.2234	-0.0074	-1.3349	0.1831	-0.0024	-0.6177	0.5373
Time (days from ses2)	0.0001	2.3405	**0.0202**	0.0001	1.8114	0.0717	0.0000	0.2947	0.7685	0.0000	-0.2274	0.8203
Group: Dys Ctrl	-0.0183	-0.8463	0.3992	0.0256	1.3504	0.1798	0.0160	0.8460	0.3995	0.0100	0.6113	0.5426
Group: Typ Ctrl	0.0214	1.3047	0.1947	0.0296	1.8934	0.0610	0.0523	3.0386	**0.0030**	0.0415	2.8221	**0.0059**
Time * Group: Dys Ctrl	0.0001	1.1297	0.2599	-0.0001	-1.5524	0.1223	-0.0001	-1.9728	**0.0497**	0.0000	-0.7066	0.4805
Time * Group: Typ Ctrl	0.0000	-0.7688	0.4428	-0.0001	-1.1410	0.2553	0.0000	-0.2051	0.8376	0.0000	0.0438	0.9651

Table S6 | LME results for change text selectivity index over time

Log Size ~ age + movement + RunNums + Raw Reading Trait + Raw Reading State*Subgroup (1 | Participant)												
	VWFA1			VWFA2			FFA1			FFA2		
	β	t	p	β	t	p	β	t	p	β	t	p
WJ LWID												
Intercept	-1.8853	-1.5024	0.1355	-1.3224	-0.8951	0.3726	3.5784	4.6577	**9.44E-06**	2.0600	2.3092	**0.0231**
WJ LWID Trait	0.0869	3.6607	**0.0004**	0.1385	4.8190	**5.70E-06**	0.0227	1.5529	0.1247	-0.0102	-0.5815	0.5626
Group: Dys Ctrl	-0.7397	-1.9364	0.0562	-0.3033	-0.6510	0.5168	-0.1328	-0.5649	0.5740	-0.1514	-0.5282	0.5990
Group: Typ Ctrl	0.2877	0.5992	0.5506	-0.1420	-0.2433	0.8084	-0.4963	-1.6803	0.0973	-0.6072	-1.6942	0.0944
WJ LWID State	0.0907	4.9589	**1.33E-06**	0.0874	4.8009	**2.76E-06**	-0.0054	-0.4913	0.6237	-0.0015	-0.1507	0.8803
Age	0.0257	0.1675	0.8674	-0.3591	-1.9231	0.0578	-0.0522	-0.5531	0.5820	0.2006	1.7464	0.0850
Movement (Mean FD)	-0.6758	-0.8491	0.3965	-0.0840	-0.1023	0.9186	-1.1852	-2.4734	**0.0139**	-0.1915	-0.4266	0.6700
# of Runs	0.2629	2.0737	**0.0390**	0.3782	2.9460	**0.0035**	0.5704	7.4951	**8.98E-13**	0.6090	8.7710	**2.45E-16**
Group: Dys Ctrl * WJ LWID State	0.0325	0.6370	0.5247	-0.0141	-0.2776	0.7815	-0.0078	-0.2550	0.7989	0.0182	0.6665	0.5058
Group: Typ Ctrl * WJ LWID State	-0.0834	-1.5323	0.1268	-0.1157	-2.1383	**0.0335**	-0.0096	-0.2958	0.7676	-0.0270	-0.9316	0.3525
WJ WA												
Intercept	-2.3207	-1.7348	0.0852	-1.9631	-1.2352	0.2193	3.6190	4.6182	**1.13E-05**	2.2286	2.4789	**0.0149**
WJ WA Trait	0.1161	2.1379	**0.0353**	0.2052	3.0546	**0.0030**	0.0331	1.0341	0.3047	-0.0326	-0.8498	0.3982
Group: Dys Ctrl	-0.4903	-1.1236	0.2644	0.0924	0.1707	0.8648	-0.0819	-0.3187	0.7509	-0.2351	-0.7612	0.4490
Group: Typ Ctrl	0.7601	1.5147	0.1336	0.4915	0.7888	0.4325	-0.3816	-1.2910	0.2012	-0.5429	-1.5262	0.1313
WJ WA State	0.0847	2.5535	**0.0113**	0.1491	4.6957	**4.43E-06**	-0.0130	-0.6838	0.4948	-0.0163	-0.9654	0.3353
Age	0.2620	1.9327	0.0567	-0.0069	-0.0413	0.9672	0.0027	0.0336	0.9733	0.1977	2.0566	**0.0433**
Movement (Mean FD)	-1.1015	-1.3260	0.1858	-0.4299	-0.5177	0.6050	-1.3169	-2.7522	**0.0063**	-0.2942	-0.6583	0.5108
# of Runs	0.3222	2.4438	**0.0151**	0.4102	3.1868	**0.0016**	0.5506	7.2722	**3.68E-12**	0.6007	8.7145	**3.54E-16**
Group: Dys Ctrl * WJ WA State	-0.0896	-1.1108	0.2678	-0.0693	-0.8984	0.3699	0.0628	1.3598	0.1753	0.0514	1.2515	0.2120
Group: Typ Ctrl * WJ WA State	-0.0787	-0.8812	0.3791	-0.1895	-2.2136	**0.0278**	-0.0123	-0.2409	0.8098	0.0425	0.9297	0.3535
WJ SRF												
Intercept	-0.6255	-0.4146	0.6793	2.4457	1.3929	0.1667	3.9915	4.5261	**1.83E-05**	1.4030	1.3728	0.1734
WJ SRF Trait	0.0215	1.5718	0.1196	0.0645	3.9812	**0.0001**	0.0050	0.6323	0.5293	-0.0114	-1.2011	0.2335
Group: Dys Ctrl	-0.9016	-2.2405	**0.0277**	-0.6041	-1.2593	0.2114	-0.1864	-0.7950	0.4294	-0.1199	-0.4278	0.6701
Group: Typ Ctrl	0.9750	1.9102	0.0596	0.1731	0.2845	0.7767	-0.3112	-1.0465	0.2991	-0.4647	-1.3070	0.1954
WJ SRF State	0.0746	5.4684	**1.11E-07**	0.0911	6.9607	**3.05E-11**	-0.0082	-1.0118	0.3127	-0.0067	-0.9127	0.3623
Age	0.2537	1.5602	0.1226	-0.2564	-1.3203	0.1903	0.0027	0.0281	0.9777	0.2479	2.1842	**0.0322**
Movement (Mean FD)	-0.7921	-0.9927	0.3216	-0.0268	-0.0338	0.9731	-1.3235	-2.7883	**0.0056**	-0.2678	-0.6011	0.5482
# of Runs	0.2359	1.8740	0.0620	0.3118	2.5452	**0.0115**	0.5673	7.5541	**6.37E-13**	0.6243	9.1122	**2.34E-17**
Group: Dys Ctrl * WJ SRF State	-0.0005	-0.0133	0.9894	-0.0394	-1.1105	0.2679	0.0355	1.6017	0.1106	0.0366	1.8531	0.0652
Group: Typ Ctrl * WJ SRF State	-0.0630	-2.6809	0.0078	-0.1082	-4.8076	**2.69E-06**	-0.0161	-1.1453	0.2533	-0.0057	-0.4518	0.6518
TOWRE SWE												
Intercept	-0.7436	-0.5643	0.5736	1.0852	0.7254	0.4698	4.0483	5.0749	**1.78E-06**	1.8407	1.9943	**0.0491**
TOWRE SWE Trait	0.0405	3.1163	**0.0025**	0.0795	5.2419	**1.06E-06**	0.0126	1.6006	0.1138	-0.0055	-0.5855	0.5600
Group: Dys Ctrl	-0.7202	-1.8440	0.0687	-0.2560	-0.5583	0.5781	-0.1442	-0.6094	0.5443	-0.1481	-0.5183	0.6058
Group: Typ Ctrl	0.6030	1.2802	0.2039	0.0525	0.0954	0.9242	-0.4674	-1.6383	0.1058	-0.6299	-1.8366	0.0703
TOWRE SWE State	0.0652	5.5274	**8.26E-08**	0.0744	6.7116	**1.33E-10**	-0.0036	-0.5071	0.6126	0.0026	0.4063	0.6849
Age	0.1251	0.8434	0.4014	-0.3320	-1.9073	0.0599	-0.0469	-0.5221	0.6033	0.2018	1.8609	0.0668
Movement (Mean FD)	-0.5980	-0.7485	0.4547	0.1469	0.1884	0.8507	-1.2489	-2.5859	**0.0102**	-0.1784	-0.3950	0.6931
# of Runs	0.2271	1.8038	0.0723	0.3169	2.6371	**0.0089**	0.5526	7.2633	**3.98E-12**	0.6076	8.7893	**2.29E-16**
Group: Dys Ctrl * TOWRE SWE State	-0.0126	-0.4265	0.6701	-0.0186	-0.6673	0.5052	-0.0068	-0.3784	0.7055	-0.0049	-0.3053	0.7604
Group: Typ Ctrl * TWORE SWE State	-0.0306	-0.8386	0.4026	-0.0598	-1.7471	0.0819	-0.0055	-0.2517	0.8015	0.0065	0.3333	0.7392
TOWRE PDE												
Intercept	-0.7895	-0.5881	0.5576	0.9239	0.5985	0.5507	4.3027	5.4956	**2.88E-07**	2.0074	2.1629	**0.0332**
TOWRE PDE Trait	0.0697	2.8630	**0.0051**	0.1315	4.6203	**1.21E-05**	0.0388	2.7343	**0.0076**	-0.0032	-0.1837	0.8547
Group: Dys Ctrl	-0.3409	-0.7991	0.4265	0.4312	0.8535	0.3958	0.0704	0.2837	0.7775	-0.1420	-0.4628	0.6449
Group: Typ Ctrl	0.1665	0.2764	0.7828	-0.7392	-1.0463	0.2981	-0.9409	-2.6826	**0.0089**	-0.7047	-1.6481	0.1033
TOWRE PDE State	0.0807	4.1725	**4.19E-05**	0.1040	5.6297	**4.98E-08**	-0.0103	-0.9066	0.3656	-0.0115	-1.1257	0.2615
Age	0.1839	1.3199	0.1904	-0.1808	-1.0973	0.2756	-0.0819	-1.0099	0.3159	0.1628	1.6275	0.1079
Movement (Mean FD)	-0.7294	-0.8906	0.3738	-0.0604	-0.0744	0.9407	-1.2371	-2.5677	**0.0107**	-0.2526	-0.5545	0.5797
# of Runs	0.3061	2.3861	**0.0177**	0.4114	3.3075	**0.0011**	0.5656	7.4833	**9.82E-13**	0.6163	8.8945	**1.13E-16**
Group: Dys Ctrl * TOWRE PDE State	-0.0880	-1.7182	0.0871	-0.1105	-2.2650	**0.0244**	0.0144	0.4759	0.6346	0.0245	0.9057	0.3661
Group: Typ Ctrl * TOWRE PDE State	-0.0669	-1.6243	0.1056	-0.1552	-3.9498	**0.0001**	-0.0118	-0.4842	0.6287	-0.0017	-0.0801	0.9362
WJ MFF												
Intercept	-2.0983	-1.4767	0.1425	-0.5412	-0.3107	0.7566	3.9955	4.8855	**4.19E-06**	1.8408	1.9337	0.0563
WJ MFF Trait	-0.0039	-0.4533	0.6516	0.0107	0.9747	0.3326	0.0051	1.0342	0.3050	-0.0033	-0.5502	0.5839
Group: Dys Ctrl	-0.8836	-2.1395	**0.0353**	-0.4592	-0.8770	0.3830	-0.1479	-0.6215	0.5364	-0.1517	-0.5260	0.6005
Group: Typ Ctrl	1.6122	3.7617	**0.0003**	1.5494	2.8516	**0.0055**	-0.2902	-1.1752	0.2441	-0.6588	-2.2016	**0.0309**
WJ MFF State	0.0356	2.8278	**0.0051**	0.0327	2.6161	**0.0094**	0.0090	1.2472	0.2136	0.0096	1.4942	0.1365
Age	0.4606	3.1794	**0.0021**	0.1669	0.9060	0.3676	-0.0027	-0.0325	0.9742	0.1862	1.8339	0.0708
Movement (Mean FD)	-1.1051	-1.3220	0.1871	-0.6675	-0.7727	0.4403	-1.2420	-2.5929	**0.0100**	-0.1256	-0.2801	0.7796
# of Runs	0.3119	2.3680	**0.0186**	0.4018	3.0223	**0.0027**	0.5529	7.3314	**2.57E-12**	0.6117	8.9110	**9.40E-17**
Group: Dys Ctrl * WJ MFF State	-0.0132	-0.4025	0.6877	-0.0180	-0.5552	0.5793	-0.0190	-1.0120	0.3126	-0.0211	-1.2645	0.2073
Group: Typ Ctrl * WJ MFF State	-0.0111	-0.5019	0.6162	-0.0530	-2.4336	**0.0157**	-0.0291	-2.3116	**0.0217**	-0.0188	-1.6776	0.0948

Table S7 | LME results for the relationship with ROI size and raw assessment state and trait

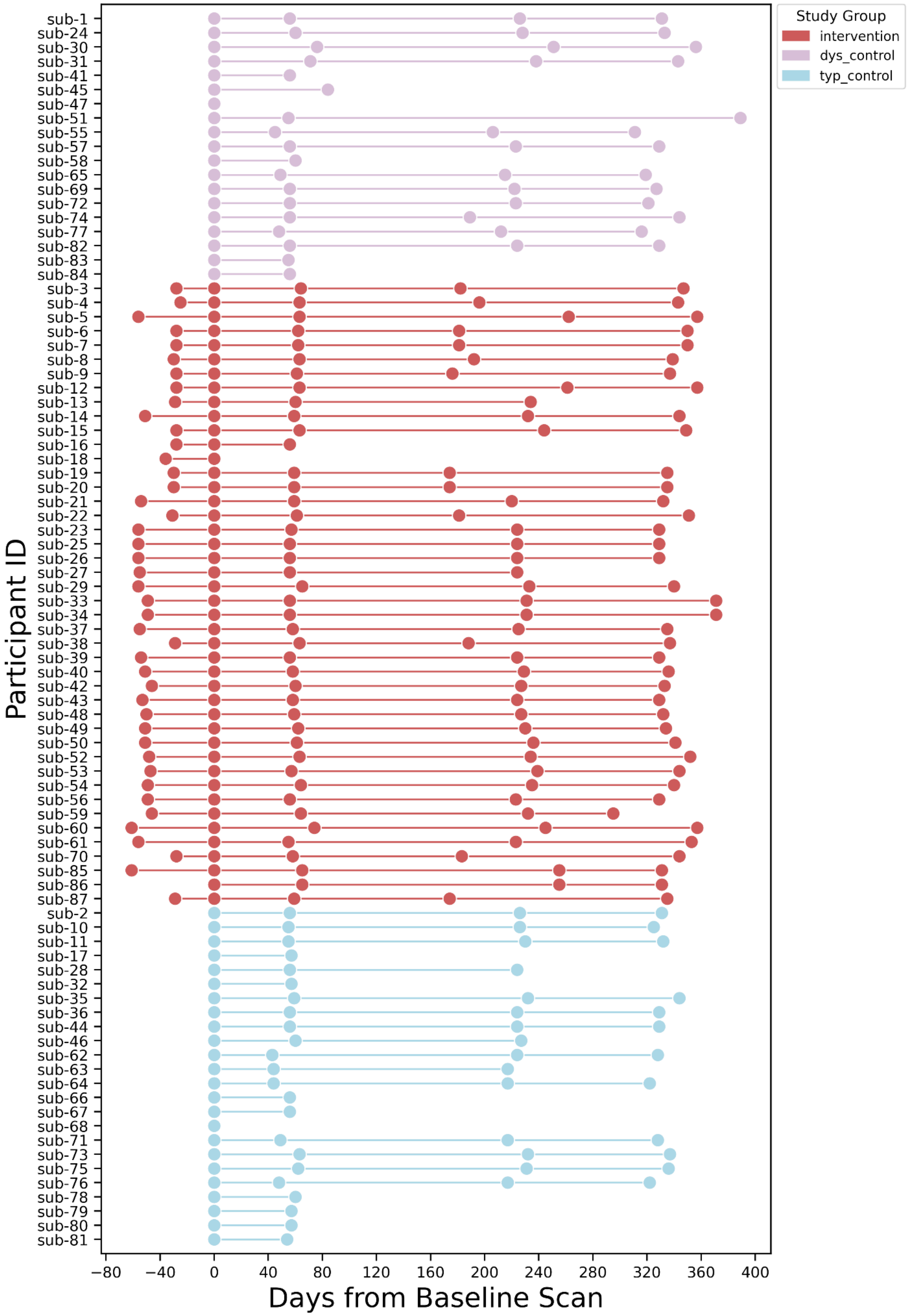

Figure S3 | Data collection timeline for each participant relative to baseline scan.

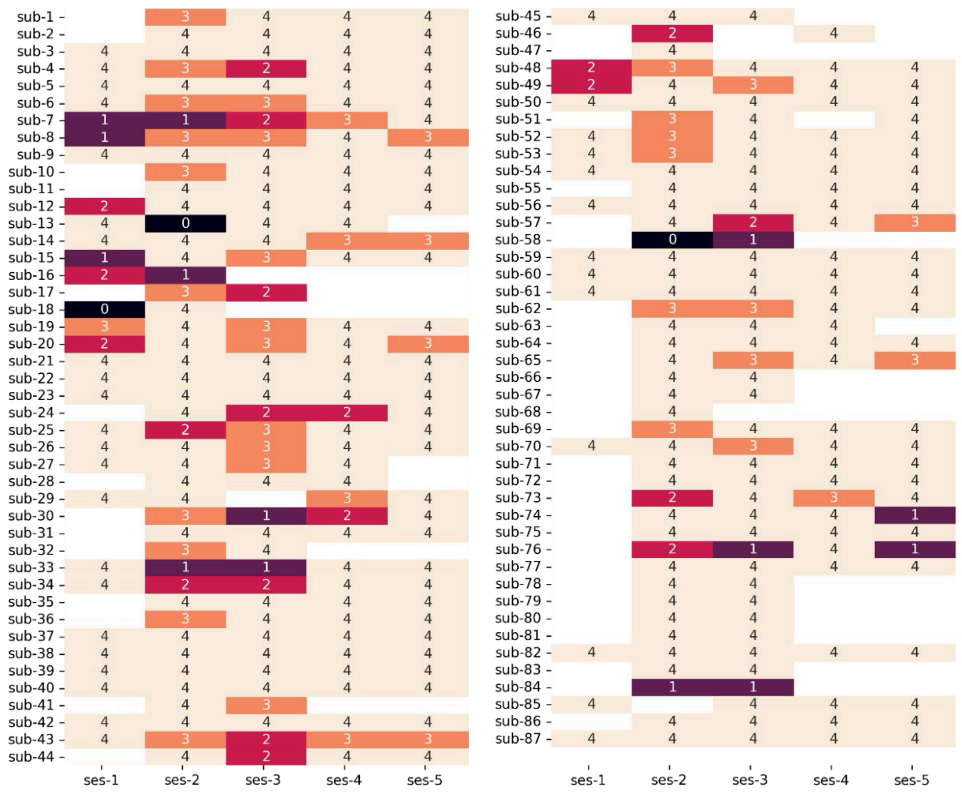

Figure S4 | Number of usable runs of the functional localizer per participant in each time point.

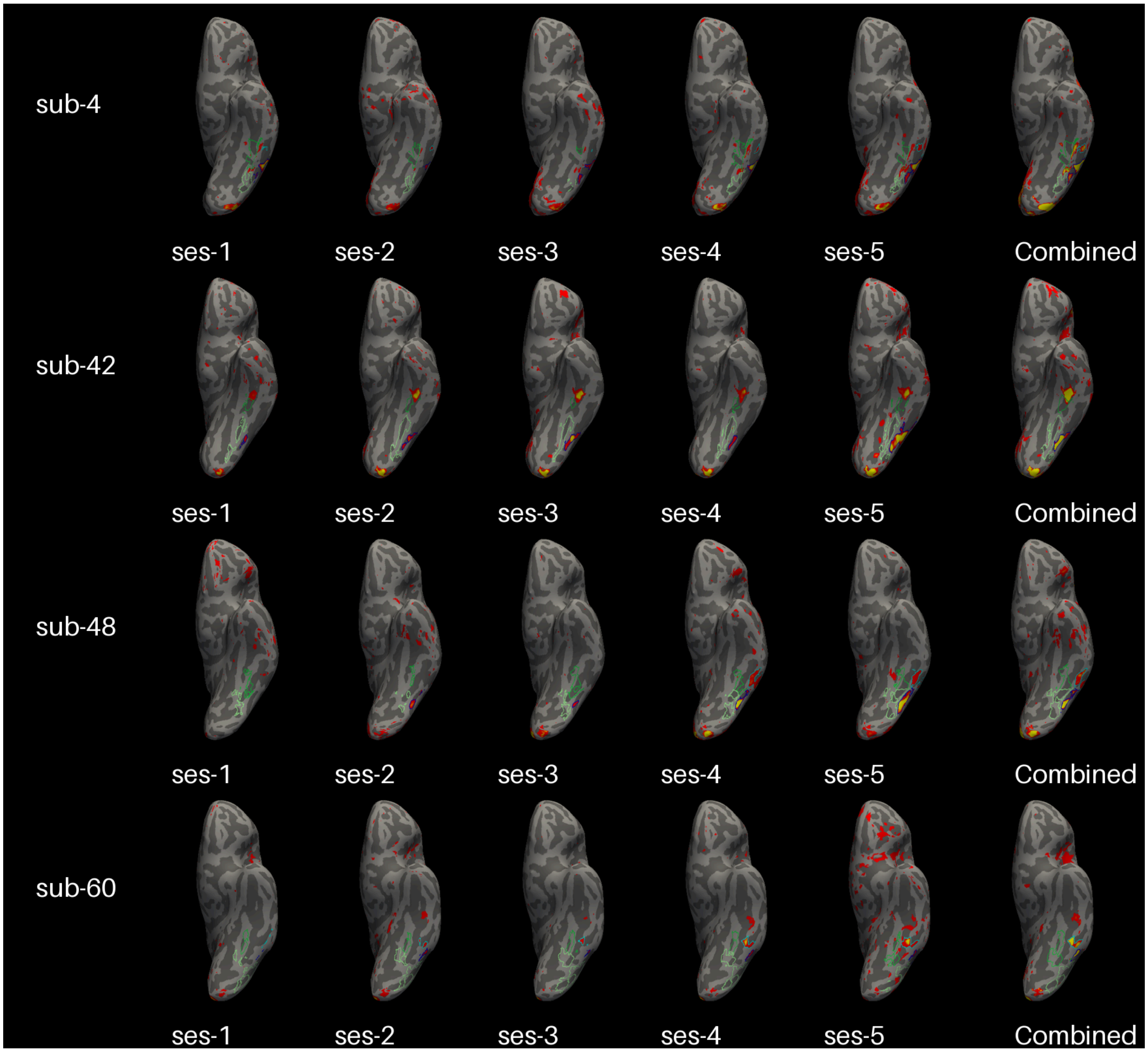

Figure S5 | ROIs drawn on the native surface for 4 sample participants for each time point and averaged across time points projected over a contrast map of text > all other categories thresholded at a t of 3. VWFA-1 is dark blue, VWFA-2 is light blue, FFA-1 is light green, and FFA-2 is dark green.

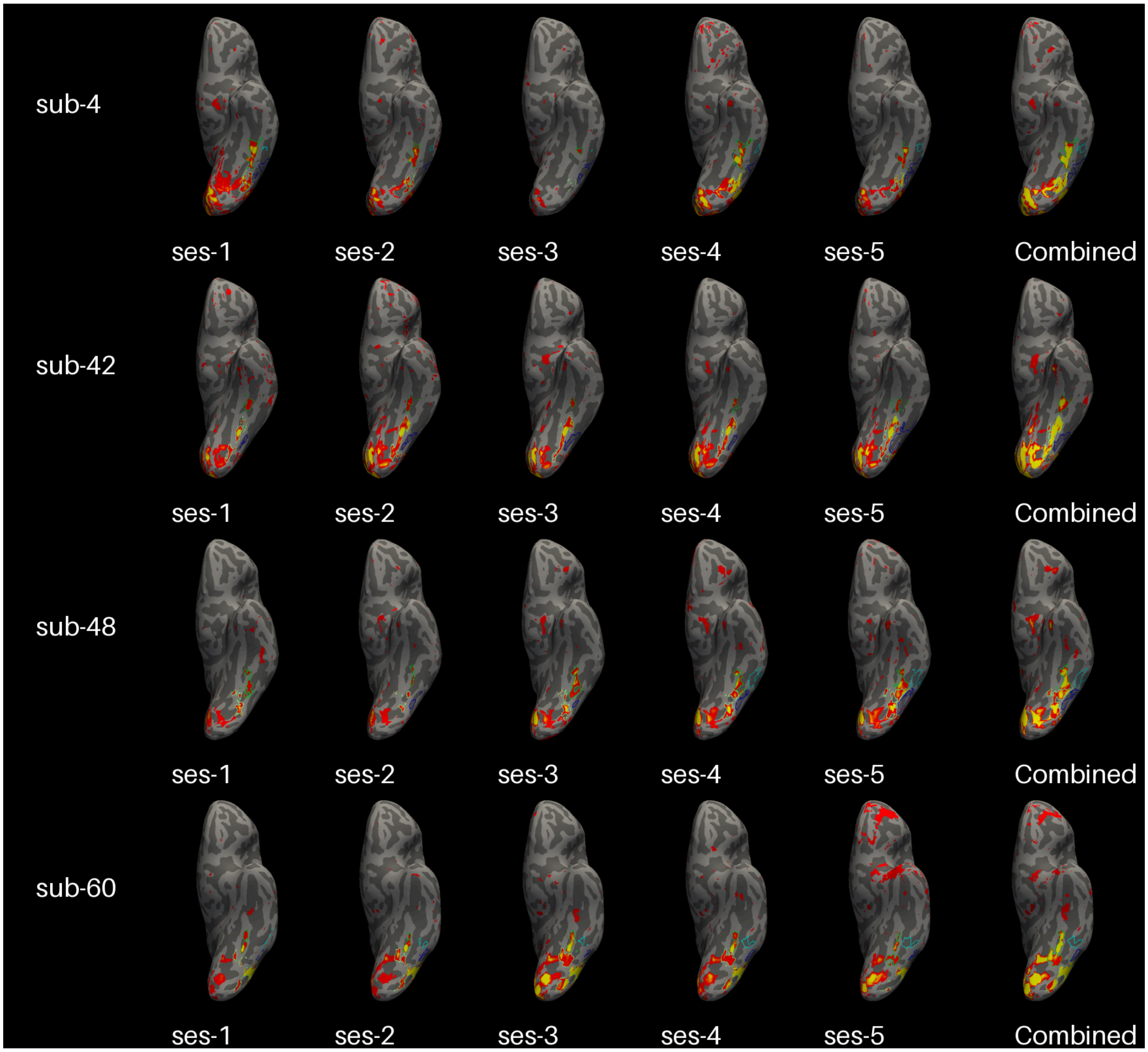

Figure S6 | ROIs drawn on the native surface for 4 sample participants for each time point and averaged across time points projected over a contrast map of faces > all other categories thresholded at a t of 3. VWFA-1 is dark blue, VWFA-2 is light blue, FFA-1 is light green, and FFA-2 is dark green.
